# Supplementary material for: An Escape Room to Orient Preclinical Medical Students to the Simulated Medical Environment
Source: MedEdPORTAL. 2022 Mar 25;18:11229. doi: 10.15766/mep_2374-8265.11229 (PMC8948100; doi:10.15766/mep_2374-8265.11229)
Supplement: Supplementary file 1 — Escape Room Simulation Guide.docxRoom Layout.pdfPatient Chart and Puzzle Template.pdfClue and Exam Findings Cards.pdfAdditional Room Resources.docxParticipant Prebriefing.pptxEscape Room Flow Chart and Codes.pdfExit Questionnaire.docxFaculty Instructions and Debriefing Guidelines.pdfCritical Actions Checklist.docxParticipant Evaluation.docxFollow-up Survey.docx [file mep_2374-8265.11229-s001.zip › A. Escape Room Simulation Guide.docx]

| Appendix A: Escape Room Simulation Guide  SIMULATION CASE TITLE: Simulation Orientation: An Escape Room Activity to Introduce Preclinical Medical Students to the Simulated Medical Environment  AUTHORS: Aimee Martin, MD, Sarah Gibbs, MS  LEARNER AUDIENCE: Preclinical Medical Students | |
| --- | --- |
| PATIENT NAME: Jay/Jameela Lawson  PATIENT AGE: 47  CHIEF COMPLAINT: Routine wellness exam  PHYSICAL SETTING: Outpatient primary care clinic | |
| Brief narrative description of case | J. Lawson has come to the primary care clinic for a routine wellness visit, requiring a complete set of vital signs and a brief physical exam.  The learners are ‘locked’ in the exam room and must solve a series of puzzles directing them to 12 tasks that must be completed to escape the room. |
| Primary Learning Objectives | Following the session, the learner will be able to:  1. Recall the layout of the simulation patient bay.  2. Demonstrate the use of basic equipment in the simulation bay.  3. Identify basic features of the simulation manikin. |
| Critical Actions | 1. Hand hygiene (wash and don gloves) 2. Communicate with patient 3. Adjust bed 4. Use phone 5. Locate the BLS algorithm in the room 6. Read patient vital signs on the monitor 7. Take manual BP 8. Take manual pulse 9. Take automated BP 10. Examine pupils with ophthalmoscope 11. Auscultate heart and lungs 12. Examine abdomen |
| Learner Preparation or Prework | Learners will complete an online module and attend a 10-minute large group didactic prebriefing describing the scenario and the rules prior to beginning the activity.  Learners are required to have the skills necessary to obtain patient vital signs and to perform a basic physical exam. |

| Initial Presentation | | | |
| --- | --- | --- | --- |
| Initial vital signs | HR 80, BP 139/80, RR 16, T 97.7 F, O2 sat 99% | | |
| Overall Setting and Appearance | The room is set up as a basic hospital room, with sink, paper towel dispenser, glove box, patient bed, rolling stool, crash cart, room phone, and patient monitor. The manikin is dressed in street clothes and is lying flat on the bed with a blanket over them. The patient bedside table is at the foot of the bed, and is the first thing the learner sees when they enter the room. The patient monitor is turned on and displaying all VS except for the BP and SPO2. The patient has a BP cuff attached to the left arm. The pulse oximeter is hanging on the monitor. | | |
| Confederates (e.g., standardized participants) and their roles in the room at case start | The manikin is operated remotely by a faculty member who voices the patient. The operator also calls into the room as the clinic nurse. | | |
| HPI | Once the learners introduce themselves:  “Hi, I’m Jay/Jameela Lawson.”  “I am here for a routine wellness exam.”  “I feel fine.” | | |
| Past Medical/Surgical History | Medications | Allergies | Family History |
| HTN | Norvasc | None | Mother-HTN, colon cancer, deceased age 84  Father-CAD, MI, deceased age 79  Brother-HTN, gout, age 43 |
| Physical Examination | | | |
| General | Awake and alert. | | |
| HEENT | PERRL, blinking, O/P clear, no cyanosis. | | |
| Neck | Heart icon taped over the right carotid pulse point. | | |
| Lungs | CTA bilaterally, equal bilateral chest rise. | | |
| Cardiovascular | Sinus rhythm, 80, no m/r/g; central and distal pulses palpable and equal. | | |
| Abdomen | NABS. QR code on left lower quadrant, below waistband. | | |
| Neurological | A&O x 4 | | |
| Skin | Warm, dry, no rashes. | | |
| Extremities | Right index finger has a diamond icon taped to it; right wrist has a heart icon taped over the radial pulse point. BP cuff on left arm labeled with a square. | | |
| GU | N/A | | |
| Psychiatric | N/A | | |

| Instructor Notes - Changes and CASE Branch Points | | |
| --- | --- | --- |
| Intervention / Time point | Change in Case | Additional Information |
| Students enter room | START TIMER | Patient responds to basic questions re demographics, CC, PMH, IF ASKED:  “Hi, I’m Jay/Jameela Lawson.”  “I’m here for a routine wellness exam.”  “I feel fine.”  “I am 47-years-old. I have high blood pressure. I take Norvasc. I don’t have any allergies.”  *If learners ask questions out of context of a patient exam, such as for clues to the escape room, say “I’m not sure what you mean?” |
| If learners do not wash and dry hands and don gloves within the first 5 minutes (i.e. do not find puzzle pieces and solve first clue/open Box 1) | Provide HINT | Patient: “I think my chart is around here somewhere. I’m puzzled as to where it could be!”  “Could you please wash your hands and wear gloves?” |
| When learners ask “How do you feel?” |  | Patient states: “My back is killing me lying like this. Can you raise the head of the bed?” |
| Once head of bed is raised  (Approx 10-15 min in) | Operator calls into room using room phone | Nurse manager (manikin operator):  “Hi, this is the clinic manager. Are you seeing Mr./Mrs. Lawson? I wanted to let you know that all of the equipment you need to complete the visit is in the room, but some of it may be locked up. You should be able to unlock it using resources found in the room. You can call me on this phone if you need help with anything, but it will take more time.” |
| If students have trouble with the blood pressure clue | Provide HINT via room phone | “There is a resource in the room to help with that clue.”  “Your answer to clue 5 is incorrect.” |
| If students have trouble taking a manual blood pressure | Provide HINT | Patient: “My blood pressure can be tricky to get sometimes. The nurse told me it was 139/80.” |
| If students have trouble with the BLS algorithm clue | Provide HINT via room phone | “There is a resource in the room to help with that clue.” |
| If students have trouble with the temp conversion | Provide HINT via room phone OR patient | “There is a resource in the room to help with that clue.”  Patient: “I saw the nurse use a thermometer-would that help you?” |
| When students place SPO2 probe on finger and attempt to obtain an automatic BP |  | Operator can manually turn on O2 sat reading and automated BP if needed. |
| If students have trouble with BMI conversions  (Approx 20 min in ) | Provide HINT via room phone | “There is a resource in the room to help with that clue.” |
| When students auscultate the chest |  | Operator can click the “Auscultation Focus” on some manikins to decrease mechanical noise. |
| If students do not examine eyes properly (do not see that the pupils are reactive) | Provide HINT | Patient: “Maybe you should dim the lights to see my eyes better-that’s what the nurse did.” |
| If students use the phone to ask for help/hints | Provide HINT via room phone | Operator can use these responses:   1. The answers to clues open locks on boxes. 2. All clues and questions can be solved/answered with resources found in the room. Look for a resource to help you. 3. Your answer to (that) clue is incorrect. 4. Other response (at operator’s discretion) |
| At 30 minutes |  | Operator calls into room:  “You have 10 minutes remaining.” |
| At 40 minutes |  | Operator calls into room:  “Time is up. The activity has ended.” |

Ideal Scenario Flow

The team enters the room and sees the first clue on the bedside table which is directing them to look for four puzzle pieces. They may also introduce themselves to the patient. Learners search the room for the puzzle pieces, as well as for any other visible resources or objects of interest (such as the locked boxes, the BMI chart on the backboard, the locked tablet on the table, the thermometer, etc.). A team member could catalogue the various boxes, locks and other items (tablet locked with a password in the form of a word). Once the pieces have been found and assembled into the patient chart, the team leader reads the patient chart and allows all members to look at it. It will become apparent that some letters appear to be randomly capitalized. Writing the capital letters down in the order that they appear, reveals the statement “WASH YOUR HANDS” with the word “HAND” outlined on the clue card. This word will open the letter combination lock on Box 1 on the bedside table. A clue card inside will instruct them to wash their hands and answer a question about handwashing, the numerical answer to which can be found on a CDC Handwashing Guide poster above the sink. The team will realize that the rest of the locked boxes placed around the room have locks with numeric combinations. Once the answer (100) is found, all boxes can be tried by multiple team members until the correct box (Box 2) is located by the sink and opened. They will find scissors and a clue suggesting they ask how the patient is feeling, and that they can use the scissors to help their patient sit up. They will clip ties holding the head of the bed flat and raise the head. At that point the Clinic Manager will call into the room and advise them they can find all of the equipment they will need for the patient visit in the room, though some of it is locked up. When the phone receiver is picked up, a clue taped to the handset will be revealed, asking them to determine the highest normal systolic blood pressure. The answer to the clue (119) can be determined from a chart attached to the crash cart (identified as Box 3). This number will open the lock on the crash cart, allowing the drawers to be opened. A team member will go through the draws and find a clue with a stethoscope, advising them to check the patient's blood pressure, as well as a second clue asking them to order four pieces of paper, each with one step of the Basic Life Support algorithm. Each paper has a number (ranging from 1-4) on the back. When the steps are ordered correctly, the order of the numbers on the back is a code (3142) that opens the only box with a four-digit lock (Box 4) The BLS algorithm is attached to the crash cart to help with this puzzle. Meanwhile another team member should be obtaining a manual BP on the patient. The box with the four-digit lock will contain a transparency with symbols taped on it in a pattern. The learners will realize that the transparency can be fitted over the patient monitor to reveal which vital sign is associated with which symbol. Learners should notice that the O2 sat probe and the patient’s finger are both marked with the same symbol (a diamond) at this point, and apply the probe. There will also be a clue card in this box asking for the patient’s temperature (visible on the patient monitor in Fahrenheit) to be converted to Celsius. The team will have to find the conversion chart on the back of the thermometer to determine the code (365) that will open the next box (Box 5). There will also be cards in Box 5 directing the students to examine the patient’s mouth and listen to the heart. Box 5 will reveal another clue asking learners to determine the patient’s weight in pounds. The team will need to find a BMI chart hidden in the room on the CPR board, and use the patient’s BMI and height provided on the patient chart (the four puzzle pieces) to determine this code (180). This answer will open the next box (Box 6) which will have cards inside directing the learners to examine the patient’s eyes and lungs. The ophthalmoscope in the room is marked with a star, as is the card directing the eye exam. The eye examination will reveal reactive pupils. The word “reactive” is the passcode to open the locked tablet. The tablet lock screen also has a star on it to connect the clues. Once the tablet is unlocked, they will see a clue advising them to perform an abdominal exam. A QR code will be found on the patients left lower abdomen. Once scanned, a Qualtrics survey will open, asking them to input the patient’s vital signs and exam findings. Upon completion of this they will be directed to a message that states “Congratulations, you have completed your patient’s clinic visit in time and have escaped the room!”

Anticipated Management Mistakes

1. *Finding clues out of order: some teams may find the clue under the phone handset before finding the puzzle pieces that make up the patient chart. This is ok, but they will eventually need to find the puzzle pieces to answer the question about the patient’s weight.*
2. *Failure of team members to share clues they have found: Frequently, a learner will find a clue, or an aid to answer a clue, but will not communicate this out loud to the team, resulting in delays. This could be addressed in the debrief.*
3. *Difficulty answering the blood pressure question: The blood pressure question is worded in such a way that learners need to be precise in answering it. They often try the number 120 and then give up on the clue when no locks are able to be opened. A systolic blood pressure of 120 is considered to be elevated according to current guidelines. The operator may need to call into the room to notify them that the answer is wrong, and/or that they need to find the resource (BP chart) in the room to help them.*
4. *Trying to take a manual BP without a stethoscope: Learners who haven't yet found the stethoscope often wonder if the manikin has some way of providing a manual blood pressure without the use of a stethoscope, and may need the operator to call in and remind them they will need a stethoscope to obtain a manual blood pressure. The patient monitor can display an automated blood pressure that does not require use of the stethoscope, and both of these methods to obtain a blood pressure should be discussed in the debrief.*
5. *Difficulty hearing Korotkoff sounds on the manikin or taking the manual BP: Learners are preclinical students with minimal experience in obtaining BPs, and none on obtaining them in manikins. The manikin operator can provide the BP to the students, if needed, and the skill can be demonstrated in the debrief.*
6. *Locking out the tablet: Learners may need to be reminded of how many attempts they have before the tablet locks (this feature could not be disabled on our tablets).*
