# Supplementary material for: An Escape Room to Orient Preclinical Medical Students to the Simulated Medical Environment
Source: MedEdPORTAL. 2022 Mar 25;18:11229. doi: 10.15766/mep_2374-8265.11229 (PMC8948100; doi:10.15766/mep_2374-8265.11229)
Supplement: Supplementary file 1 — Escape Room Simulation Guide.docxRoom Layout.pdfPatient Chart and Puzzle Template.pdfClue and Exam Findings Cards.pdfAdditional Room Resources.docxParticipant Prebriefing.pptxEscape Room Flow Chart and Codes.pdfExit Questionnaire.docxFaculty Instructions and Debriefing Guidelines.pdfCritical Actions Checklist.docxParticipant Evaluation.docxFollow-up Survey.docx [file mep_2374-8265.11229-s001.zip › C. Patient Chart and Puzzle Template.pdf]

patient name: j. la**W**son

dob: 2/7/73 ht: 5'11" bmi: 25

chief concern/re**A**son for visit: follow up

pa**S**t medical **H**istory:
h**Y**pertension

medications: n**O**rvasc allergies: none

family history:
mother - deceased age 84 (hypertension, colon cancer)
father - deceased age 79 (myocardial infarction)
brother - 43 (hypertension, go**U**t)

social history:
ma**R**ried, lives with wife and one child. reports social alco**H**ol use. no tobacco use.

physical ex**A**m:
ge**N**eral appearance
eyes
mouth/throat
heart
lungs
ab**D**omen
pul**S**es

All images and graphics are author-owned.
