# Supplementary material for: An Escape Room to Orient Preclinical Medical Students to the Simulated Medical Environment
Source: MedEdPORTAL. 2022 Mar 25;18:11229. doi: 10.15766/mep_2374-8265.11229 (PMC8948100; doi:10.15766/mep_2374-8265.11229)
Supplement: Supplementary file 1 — Escape Room Simulation Guide.docxRoom Layout.pdfPatient Chart and Puzzle Template.pdfClue and Exam Findings Cards.pdfAdditional Room Resources.docxParticipant Prebriefing.pptxEscape Room Flow Chart and Codes.pdfExit Questionnaire.docxFaculty Instructions and Debriefing Guidelines.pdfCritical Actions Checklist.docxParticipant Evaluation.docxFollow-up Survey.docx [file mep_2374-8265.11229-s001.zip › E. Additional Room Resources.docx]

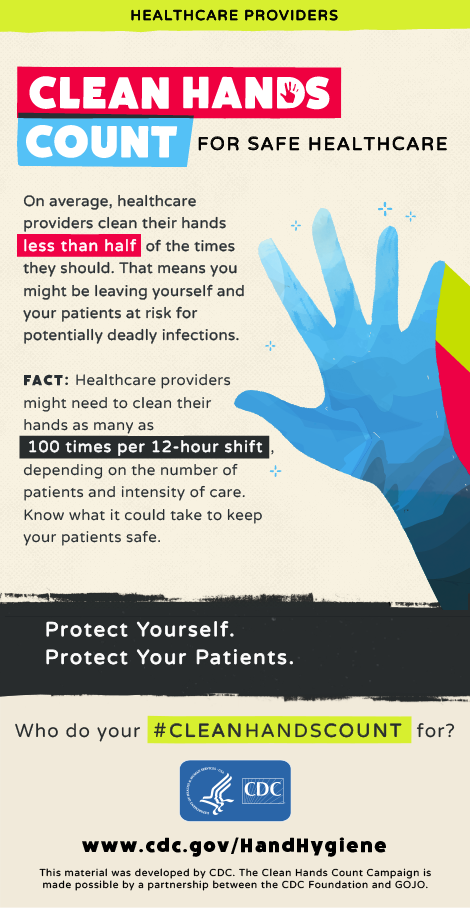


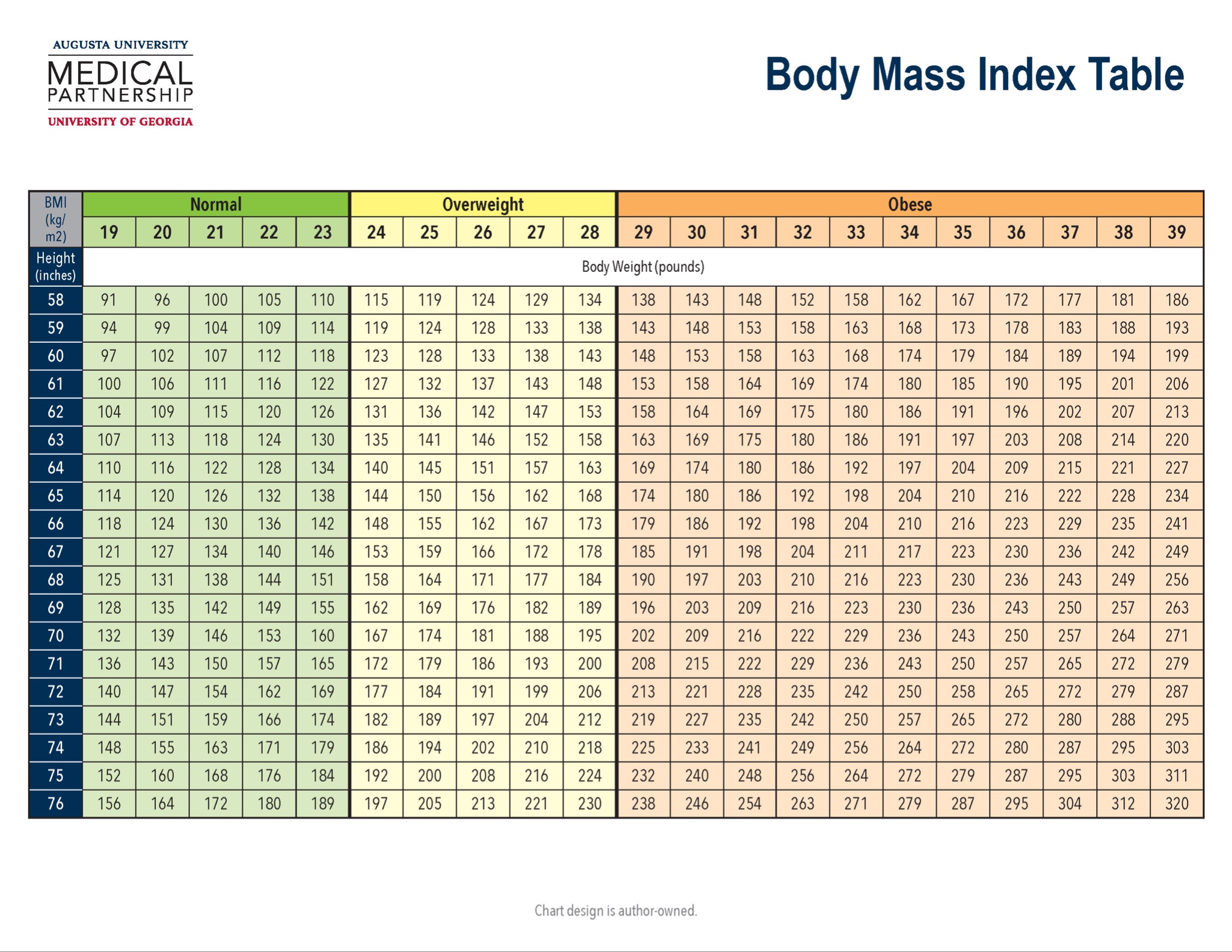
**TEMPERATURE CONVERSION CHART**

| **°C** | **°F** |
| --- | --- |
| 36.4 | 97.6 |
| 36.5 | 97.7 |
| 37.0 | 98.6 |
| 37.4 | 99.4 |
| 37.6 | 99.6 |
| 38.1 | 100.6 |
| 39.0 | 102.2 |
| 40.0 | 104.0 |
| 41.0 | 105.8 |

Image is author owned.

**For AHA BLS Algorithm resource, please look up the latest version at the American Heart Association.**

**For AHA Blood Pressure Guidelines Resource, please look up the latest version at the American Heart Association.**
