# Supplementary material for: An Escape Room to Orient Preclinical Medical Students to the Simulated Medical Environment
Source: MedEdPORTAL. 2022 Mar 25;18:11229. doi: 10.15766/mep_2374-8265.11229 (PMC8948100; doi:10.15766/mep_2374-8265.11229)
Supplement: Supplementary file 1 — Escape Room Simulation Guide.docxRoom Layout.pdfPatient Chart and Puzzle Template.pdfClue and Exam Findings Cards.pdfAdditional Room Resources.docxParticipant Prebriefing.pptxEscape Room Flow Chart and Codes.pdfExit Questionnaire.docxFaculty Instructions and Debriefing Guidelines.pdfCritical Actions Checklist.docxParticipant Evaluation.docxFollow-up Survey.docx [file mep_2374-8265.11229-s001.zip › G. Escape Room Flow Chart and Codes.pdf]

AUGUSTA UNIVERSITY
MEDICAL PARTNERSHIP
UNIVERSITY OF GEORGIA

Escape Room Activity

FLOW CHART & CODES

Lock (in order of Flow Chart)	Code	Where will lock be placed?
**Lock 1**	**HAND**	Box 1
**Lock 2**	**100**	Box 2 (with scissors and Clue card 4)
**Lock 3**	**119**	Crash Cart (Box 3)
**Lock 4**	**3142**	Box 4 (with transparency)
**Lock 5**	**365**	Box 5 (mouth & heart sounds)
**Lock 6**	**180**	Box 6 (lung sounds & pupils)
**iPad**	**"reactive"**	On iPad lock screen. iPad is located on the table on the side of the room.

CLUE CARD	helps participants advance to the next step or puzzle.
EXAM FINDINGS CARD	has blanks on it for participants to write in exam findings. These findings will be used at the end in the Exit Questionnaire as the final step to escape the room.

All images and graphics are author-owned.

Clue / Exam Findings Cards	Location	Question/Statement	Action	Result	Operator Actions & Possible Hints
CLUE CARD<br>1	Table at the foot of bed.<br>Puzzle piece locations:<br>1. Wire basket on room headboard<br>2. On tray under patient bed<br>3. Taped under large table<br>4. Taped to back of door	There was a chart on the door,<br>But now it's broken into four.<br>It's a puzzle, I presume.<br>Find the pieces in the room.<br>"W __ __ __ __ __ __ __ __ __ __ __ __ S!"	Students find 4 puzzle pieces that form a patient chart<br><br>Students fill in the blanks: "WASH YOUR HANDS"	Students open Lock 1 with code **HAND** and find Clue Card 2	***IF ASKED:***<br>*"Hi, I'm Jay/Jameela Lawson."*<br>*"I have high blood pressure.*<br>*"I take Norvasc."*<br>*"I'm 47 years old."*<br>*"I don't have any allergies."*<br>*"I feel fine."*<br><br>***HINT: PATIENT SAYS:*** *"I think my chart is around here somewhere. I'm puzzled as to where it would be."*
CLUE CARD<br>2	Box 1	Go wash your hands and get some gloves,<br>That's what everyone does.<br>Answer this question and don't get miffed,<br>How many times will you wash your hands on a 12-hour shift?	Students use CDC handwashing poster above sink.	Students find the answer is **100**, which is the clue to the Box 2.	***HINT: PATIENT SAYS:*** *"Could you please wash your hands and wear gloves?"*
CLUE CARD<br>3	Box 2:<br>• Clue Card 3<br>• Scissors	"Ask your patient how they feel,<br>You might need to raise their head,<br>With these scissors clip a tie,<br>It is attached to something red"	Students look around the room for the **RED** bed lever.	Students find **RED** bed lever and use scissors to clip the zip tie on the handle. Students raise the head of bed.	***ONCE BED IS RAISED, OPERATOR ACTING AS CLINIC MANAGER CALLS OVER THE PHONE:***<br>*"Hi, this is the Clinic Manager. Are you seeing Mr/Ms Lawson? I wanted to let you know that all the equipment you need is in the room, but some of it may be locked up. You should be able to get it out using resources in the room. You can all me on this phone if you need help, but it will take more time."*
CLUE CARD<br>4	Under phone	What is the highest systolic blood pressure that is considered normal?	Students use BP chart located on pull-out drawer on crash cart (Box 3).	Answer: **119**<br>This code opens Lock 3 on crash cart (Box 3).	***HINT:*** *"Your answer to Clue 4 is incorrect. Look for a resource to help you answer the question."*

Clue / Exam Findings Cards	Location	Question/Statement	Action	Result	Operator Actions & Possible Hints
EXAM FINDINGS CARD 1	3rd drawer of crash cart (Box 3):<br>• Exam Findings Card 1<br>• Stethoscope with ■ on it	"After that refresher, Don't let down your guard, Take your patient's pressure, And write it on the card"	Students find ■ on manikin BP cuff	Students take manual BP and write result on Exam Findings Card 1	***HINT: PATIENT SAYS:*** *"My blood pressure can be tricky to get sometimes. The nurse told me it was 139/80."*
CLUE CARD 5	Bottom drawer of crash cart (Box 3):<br>• Clue Card 5<br>• BLS Steps	Place the statements in the correct order for Basic Life Support	Students place steps in correct order. Can use BLS algorithm attached to crash cart handle.	Students find numbers on the back of steps make a code = **3142**.	***HINT:*** *"There is a resource in the room to help with that clue."*
CLUE CARD 6	Box 4 - On rolling stool next to patient monitor:<br>• Clue Card 6<br>• Exam Findings Card 2<br>• Monitor Transparency	Convert patient's temperature to Celsius	Students find temp in F on monitor and convert to C (round to nearest tenth). Can use temp conversion chart on back of thermometer, which is in the basket on the room headboard.	C = 36.5<br>**365** is the next code, which will open Box 4.	***HINT:*** *"There is a resource in the room to help with that clue."*<br><br>***HINT: PATIENT SAYS:*** *"I saw the nurse use the thermometer in the basket--would that help you?"*
EXAM FINDINGS CARD 2	Box 4 - On rolling stool next to patient monitor:<br>• Clue Card 6<br>• Exam Findings Card 2<br>• Monitor Transparency	Transparency with ♦♥♣♠ and ■	Students place transparency over monitor screen, then students tap on ■	Students fill in Exam Findings Card #2 with vitals corresponding to<br>♦=_ ♥=_<br>♣=_ ♠=_<br>■=___	***INSTRUCTION:*** Operator can manually turn on SpO_2 and heart rate and blood pressure if needed.
CLUE CARD 7	Box 5:<br>• Clue Card 7<br>• Exam Findings Card 3<br>• Exam Findings Card 4	What is the patient's weight in pounds? (chart is on the back of CPR board).They will also need the patient's Height and BMI found on the patient chart puzzle pieces.	Students use BMI chart	The weight in pounds is **180** and is the next code, which will open Box 6	***HINT:*** *"There is a resource in the room to help with that clue."*

Clue / Exam Findings Cards	Location	Question/Statement	Action	Result	Operator Actions & Possible Hints
EXAM FINDINGS CARD 3	Box 5 :<br>• Clue Card 7<br>• Exam Findings Card 3<br>• Exam Findings Card 4	Determine the following about the patient's mouth (circle one)	Students look in patient's mouth.	Students circle the finding on Exam Findings Card 3.	***PATIENT SAYS:*** *"Aahhhh"*
EXAM FINDINGS CARD 4	Box 5 :<br>• Clue Card 7<br>• Exam Findings Card 3<br>• Exam Findings Card 4	Determine the following about the patient's heart (circle one)	Students listen to heart sounds.	Students circle the finding on Exam Findings Card 4.	Operator clicks "Auscultation focus" while students are listening to the heart.
EXAM FINDINGS CARD 5	Box 6:<br>• Clue Card 8<br>• Exam Findings Card 5	Determine the following about the patient's lung's (circle one)	Students listen to lung sounds.	Students circle the finding on Exam Findings Card 5.	Operator clicks "Auscultation focus" while students are listening to the lungs.
CLUE CARD 8	Box 6:<br>• Clue Card 8<br>• Exam Findings Card 5	Examine pupils (circle one) ★	Students find ophthalmo-scope on wall or penlight with ★<br>Tablet lock screen has same symbol and *"This iPad locks after 10 wrong passcode attempts. Choose wisely!"*	The answer to the examine pupil clue is:<br>"**reactive**" which opens tablet	***HINT:***<br>***PATIENT SAYS:*** *"Maybe you should dim the lights to see my eyes."*
CLUE CARD 9	Tablet home screen	"Perform abdominal exam"	Students perform abdominal exam	Students find QR code on lower abdomen and scan	***HINT: PATIENT SAYS:*** *"Did you check my lower abdomen?"*
**Exit Questionnaire**	On electronic device (tablet) on left side of the room.	Fill in all results from patient exam.	Students submit the survey. (any answers accepted)	Automatic Message: *"Congratulations! You have completed the escape room."*	

The final step is an online questionnaire where students will fill in all of their findings from the escape room to complete the challenge.
