# Supplementary material for: An Escape Room to Orient Preclinical Medical Students to the Simulated Medical Environment
Source: MedEdPORTAL. 2022 Mar 25;18:11229. doi: 10.15766/mep_2374-8265.11229 (PMC8948100; doi:10.15766/mep_2374-8265.11229)
Supplement: Supplementary file 1 — Escape Room Simulation Guide.docxRoom Layout.pdfPatient Chart and Puzzle Template.pdfClue and Exam Findings Cards.pdfAdditional Room Resources.docxParticipant Prebriefing.pptxEscape Room Flow Chart and Codes.pdfExit Questionnaire.docxFaculty Instructions and Debriefing Guidelines.pdfCritical Actions Checklist.docxParticipant Evaluation.docxFollow-up Survey.docx [file mep_2374-8265.11229-s001.zip › H. Exit Questionnaire.docx]

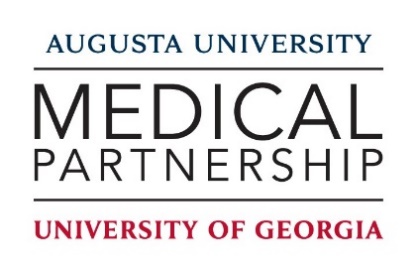


ESCAPE ROOM ACTIVITY

EXIT QUESTIONNAIRE

Q1 What vital sign parameter is represented by the spade symbol? ♠

- Automated blood pressure
- Heart rate
- Manual blood pressure
- Oxygen saturation (SPO2)
- Respiratory rate
- Temperature

Q2 What vital sign parameter is represented by the heart symbol? ♥

- Automated blood pressure
- Heart rate
- Manual blood pressure
- Oxygen saturation (SPO2)
- Respiratory rate
- Temperature

Q3 What vital sign parameter is represented by the club symbol? ♣

- Automated blood pressure
- Heart Rate
- Manual blood pressure
- Oxygen saturation (SPO2)
- Respiratory rate
- Temperature

Q4 What vital sign parameter is represented by the diamond symbol? ♦

- Automated blood pressure
- Heart rate
- Manual blood pressure
- Oxygen saturation (SPO2)
- Respiratory rate
- Temperature

Q5 What vital sign parameter is represented by the square symbol? ■

- Automated blood pressure
- Heart rate
- Manual blood pressure
- Oxygen saturation (SPO2)
- Respiratory rate
- Temperature

Q6 Manual Pulse:

________________________________________________________________

Q7 Manual BP:

________________________________________________________________

Q8 Automated BP:

________________________________________________________________

Q9 Oxygen Saturation:

________________________________________________________________

Q10 Respiratory rate:

________________________________________________________________

Q11 Heart rate:

________________________________________________________________

Q12 Temperature:

________________________________________________________________

Q13 Pupils:

- Reactive
- Unreactive
- Right pupil dilated
- Left pupil dilated

Q14 Mouth/Throat

- Abnormal mucosal or skin coloration present
- Airway clear
- Foreign object present
- Secretions present

Q15 Lung sounds:

- No lung sounds
- Clear (normal) lung sounds

Q16 Heart sounds:

- No heart sounds
- Regular rhythm
- Irregular rhythm

Q17 Abdominal exam:

- Tender when palpated
- Non-tender when palpated

Congratulations! You have completed the Escape Room!
