# Supplementary material for: An Escape Room to Orient Preclinical Medical Students to the Simulated Medical Environment
Source: MedEdPORTAL. 2022 Mar 25;18:11229. doi: 10.15766/mep_2374-8265.11229 (PMC8948100; doi:10.15766/mep_2374-8265.11229)
Supplement: Supplementary file 1 — Escape Room Simulation Guide.docxRoom Layout.pdfPatient Chart and Puzzle Template.pdfClue and Exam Findings Cards.pdfAdditional Room Resources.docxParticipant Prebriefing.pptxEscape Room Flow Chart and Codes.pdfExit Questionnaire.docxFaculty Instructions and Debriefing Guidelines.pdfCritical Actions Checklist.docxParticipant Evaluation.docxFollow-up Survey.docx [file mep_2374-8265.11229-s001.zip › J. Critical Actions Checklist.docx]

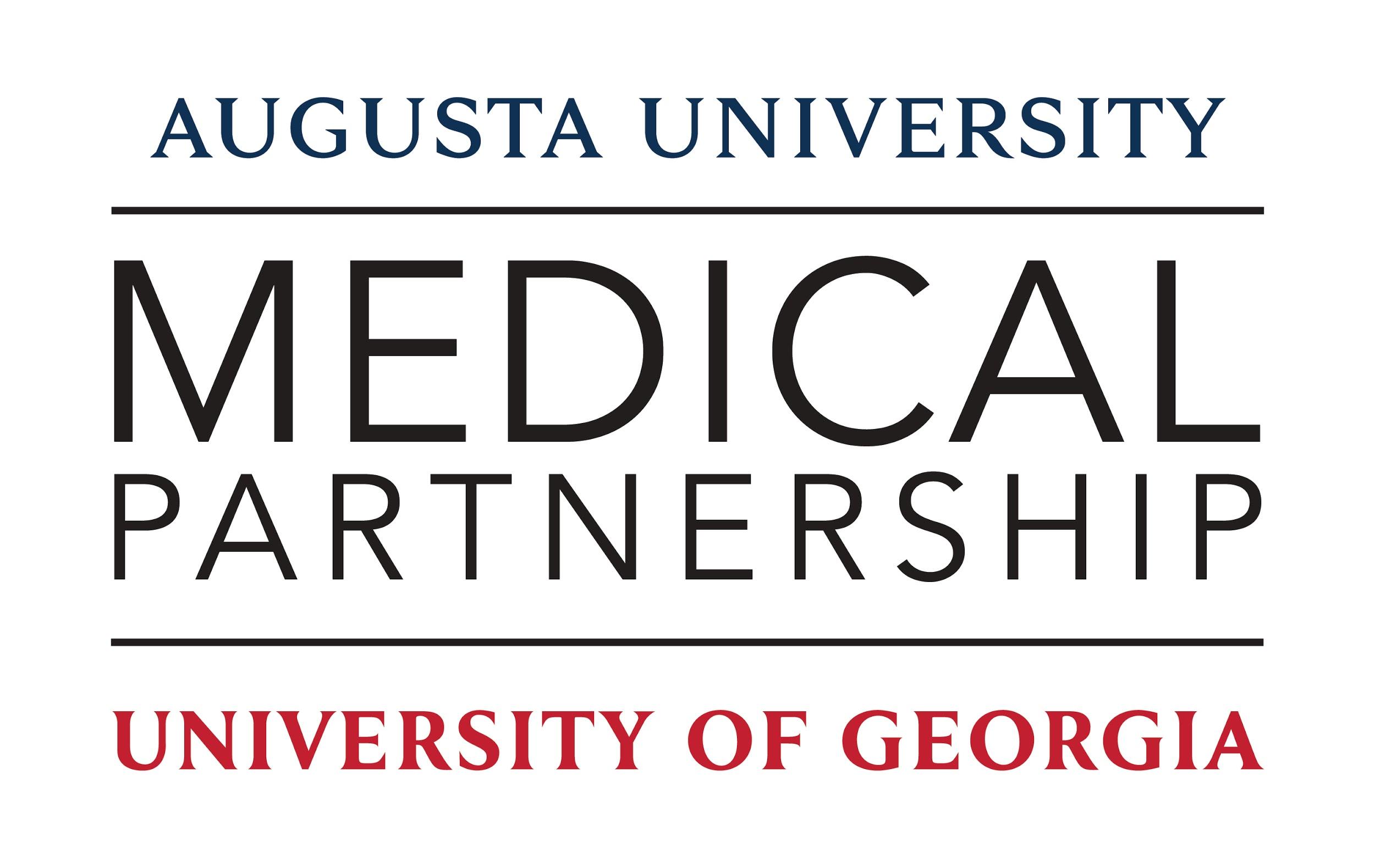
ESCAPE ROOM ACTIVITY

**CRITICAL ACTIONS CHECKLIST**

| Task | Completed | Not Completed *(Adds one minute to time for each if not completed)* | Hint Provided *(Adds one minute to time for each)* |
| --- | --- | --- | --- |
| Hand hygiene (wash and don gloves) |  |  |  |
| Communicate with patient |  |  |  |
| Adjust bed |  |  |  |
| Use phone |  |  |  |
| Locate the BLS algorithm (crash cart) |  |  |  |
| Read patient vital signs (monitor) |  |  |  |
| Take manual BP |  |  |  |
| Take manual Pulse |  |  |  |
| Take automated BP |  |  |  |
| Examine pupils with ophthalmoscope |  |  |  |
| Auscultate heart and lungs |  |  |  |
| Examine abdomen |  |  |  |
