# Supplementary material for: An Escape Room to Orient Preclinical Medical Students to the Simulated Medical Environment
Source: MedEdPORTAL. 2022 Mar 25;18:11229. doi: 10.15766/mep_2374-8265.11229 (PMC8948100; doi:10.15766/mep_2374-8265.11229)
Supplement: Supplementary file 1 — Escape Room Simulation Guide.docxRoom Layout.pdfPatient Chart and Puzzle Template.pdfClue and Exam Findings Cards.pdfAdditional Room Resources.docxParticipant Prebriefing.pptxEscape Room Flow Chart and Codes.pdfExit Questionnaire.docxFaculty Instructions and Debriefing Guidelines.pdfCritical Actions Checklist.docxParticipant Evaluation.docxFollow-up Survey.docx [file mep_2374-8265.11229-s001.zip › K. Participant Evaluation.docx]

ESCAPE ROOM ACTIVITY

**PARTICIPANT EVALUATION**
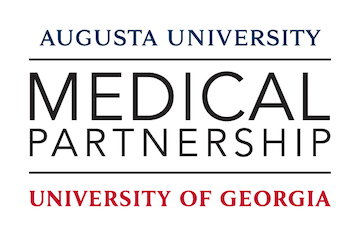


Thank you for providing feedback on the Simulation Escape Room Activity. Your participation in this survey is completely voluntary and all of your responses are anonymous.

Q1.For each of the following, please describe how confident you feel in your ability to:

|  | **Not confident at all (1)** | **Slightly confident (2)** | **Moderately confident (3)** | **Very confident (4)** | **Extremely confident (5)** |
| --- | --- | --- | --- | --- | --- |
| **Perform hand hygiene in the simulated patient room** | ◯ | ◯ | ◯ | ◯ | ◯ |
| **Adjust the patient bed** | ◯ | ◯ | ◯ | ◯ | ◯ |
| **Communicate with the patient simulator** | ◯ | ◯ | ◯ | ◯ | ◯ |
| **Use the in-room phone** | ◯ | ◯ | ◯ | ◯ | ◯ |
| **Locate the BLS (Basic Life Support) algorithm in the room** | ◯ | ◯ | ◯ | ◯ | ◯ |
| **Read the patient vital signs on the monitor** | ◯ | ◯ | ◯ | ◯ | ◯ |
| **Obtain a manual pulse on the patient simulator** | ◯ | ◯ | ◯ | ◯ | ◯ |
| **Obtain a manual blood pressure on the patient simulator** | ◯ | ◯ | ◯ | ◯ | ◯ |
| **Obtain an automated blood pressure on the monitor** | ◯ | ◯ | ◯ | ◯ | ◯ |
| **Examine the patient simulator's pupils** | ◯ | ◯ | ◯ | ◯ | ◯ |
| **Examine the patient simulator's heart and lungs** | ◯ | ◯ | ◯ | ◯ | ◯ |
| **Examine the patient simulator's abdomen** | ◯ | ◯ | ◯ | ◯ | ◯ |

Q2. What did you find helpful about the pre-briefing session?

Q3. What did you find helpful about the debriefing session?

Q4. What did you like most about the Intro to Simulation Escape Room activity?

Q5. Do you have any suggestions for improving the Intro to Simulation Escape Room activity?

Q6. Provide facilitator feedback:

|  | **Extremely Ineffective (1)** | **Mostly Ineffective (2)** | **Somewhat Effective (3)** | **Mostly Effective (4)** | **Extremely Effective (5)** |
| --- | --- | --- | --- | --- | --- |
| **The facilitator structured the debriefing in an organized way.** | ◯ | ◯ | ◯ | ◯ | ◯ |
| **The facilitator focused on learning and not on making people feel bad about making mistakes.** | ◯ | ◯ | ◯ | ◯ | ◯ |
| **The facilitator provoked in-depth discussions that led me to reflect on my or my team's performance.** | ◯ | ◯ | ◯ | ◯ | ◯ |
| **The facilitator provided concrete feedback on my performance or that of my team based on their honest and accurate view.** | ◯ | ◯ | ◯ | ◯ | ◯ |
| **The facilitator helped me see how to improve or how to sustain good performance.** | ◯ | ◯ | ◯ | ◯ | ◯ |

Q7. Please provide any additional feedback for your facilitator.
