# Supplementary material for: An Escape Room to Orient Preclinical Medical Students to the Simulated Medical Environment
Source: MedEdPORTAL. 2022 Mar 25;18:11229. doi: 10.15766/mep_2374-8265.11229 (PMC8948100; doi:10.15766/mep_2374-8265.11229)
Supplement: Supplementary file 1 — Escape Room Simulation Guide.docxRoom Layout.pdfPatient Chart and Puzzle Template.pdfClue and Exam Findings Cards.pdfAdditional Room Resources.docxParticipant Prebriefing.pptxEscape Room Flow Chart and Codes.pdfExit Questionnaire.docxFaculty Instructions and Debriefing Guidelines.pdfCritical Actions Checklist.docxParticipant Evaluation.docxFollow-up Survey.docx [file mep_2374-8265.11229-s001.zip › L. Follow-up Survey.docx]

ESCAPE ROOM ACTIVITY

FOLLOW UP SURVEY
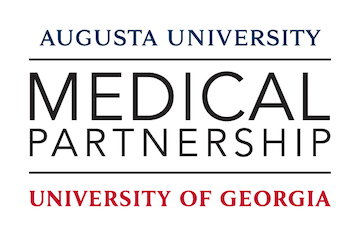


We would appreciate your feedback on introductory activity to the Simulation Center. We would like to assess your opinion as to whether the Escape Room activity was helpful prior to participating in your first simulated patient case.

Q1 Regarding the Intro to Simulation Escape Room Activity:

|  | **Yes, highly effective (1)** | **Yes, very effective (2)** | **Maybe effective (3)** | **No, not very effective (4)** | **No, definitely not effective (5)** |
| --- | --- | --- | --- | --- | --- |
| **Did you find the Escape Room to be effective in preparing you for participating in a simulated patient scenario in the Simulation Center?** | ◯ | ◯ | ◯ | ◯ | ◯ |
| **Did you find the Escape Room to be effective for acclimating you to the simulated patient room?** | ◯ | ◯ | ◯ | ◯ | ◯ |
| **Did you find the Escape Room to be effective for acclimating you to the simulated patient manikin?** | ◯ | ◯ | ◯ | ◯ | ◯ |
| **Did you find the Escape Room debriefing session to be effective for further discussion of the simulated patient room and manikin?** | ◯ | ◯ | ◯ | ◯ | ◯ |
| **Did you find the Escape Room debriefing session to be effective for introducing you to aspects of teamwork?** | ◯ | ◯ | ◯ | ◯ | ◯ |

Q2 Is there anything that you would like to change about the Escape Room activity, in order to better prepare you for participating in a simulated patient case?
