# Supplementary material for: An Escape Room to Orient Preclinical Medical Students to the Simulated Medical Environment
Source: MedEdPORTAL. 2022 Mar 25;18:11229. doi: 10.15766/mep_2374-8265.11229 (PMC8948100; doi:10.15766/mep_2374-8265.11229)
Supplement: Supplementary file 1 — Escape Room Simulation Guide.docxRoom Layout.pdfPatient Chart and Puzzle Template.pdfClue and Exam Findings Cards.pdfAdditional Room Resources.docxParticipant Prebriefing.pptxEscape Room Flow Chart and Codes.pdfExit Questionnaire.docxFaculty Instructions and Debriefing Guidelines.pdfCritical Actions Checklist.docxParticipant Evaluation.docxFollow-up Survey.docx [file mep_2374-8265.11229-s001.zip › B. Room Layout.pdf]

AUGUSTA UNIVERSITY
MEDICAL PARTNERSHIP
UNIVERSITY OF GEORGIA

ESCAPE ROOM ACTIVITY

ROOM LAYOUT

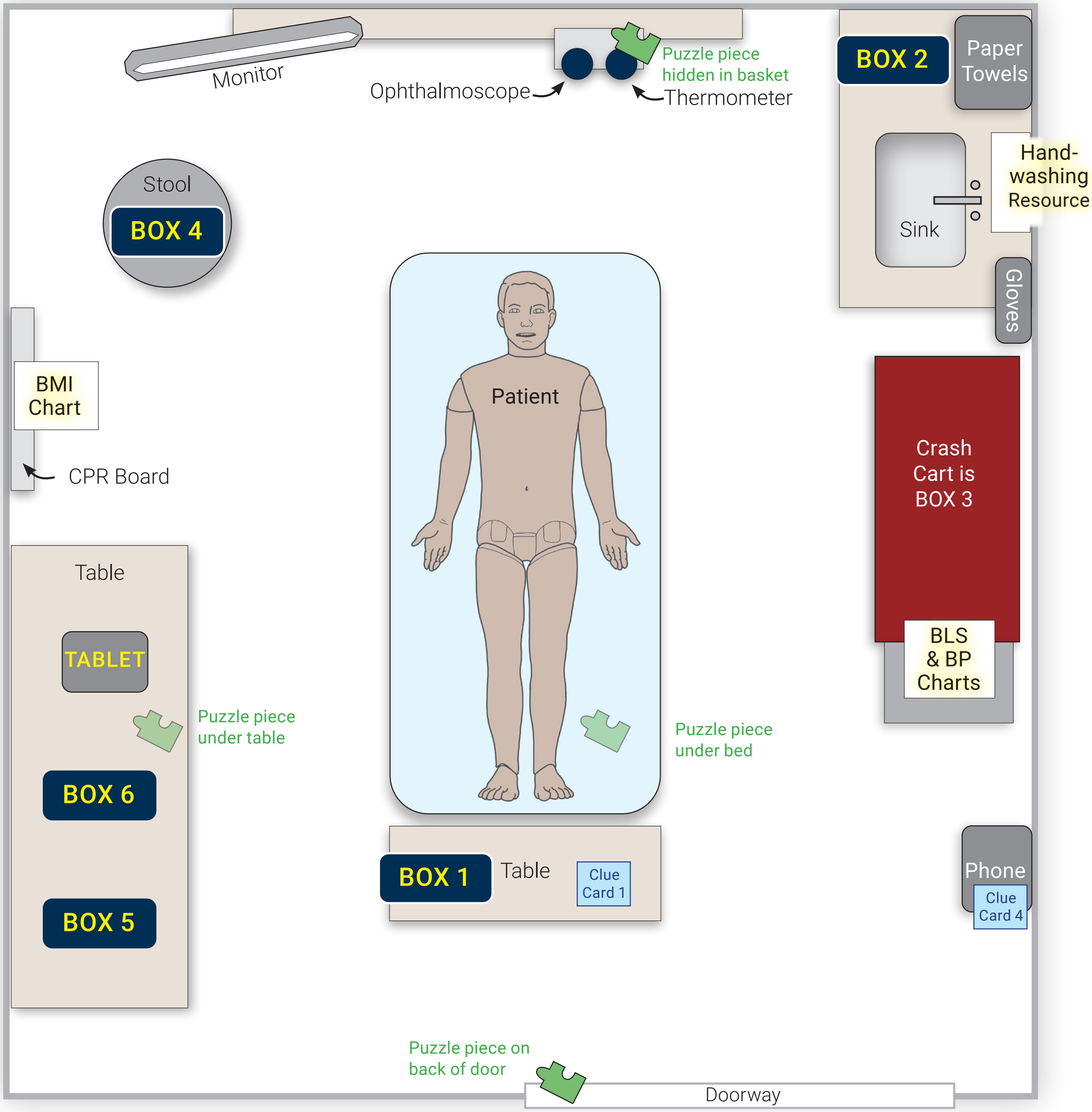

All images and graphics are author-owned.

ROOM EQUIPMENT AND MATERIALS

Sim Room Equipment

- High-Fidelity Manikin
- Patient Monitor with SpO_2 Probe
- Crash Cart
- Ophthalmoscope or penlight
- Thermometers with no batteries (with temp conversion chart affixed)
- Large Table
- Over-bed table
- Standard blood pressure cuff
- Stethoscope
- Stool/chair
- Patient bed with pillow and blanket
- Dry erase board and marker

Supplies

- Transparent tape or Tegaderm
- Zip Ties, used to lock the head of the bed in a flat position
- Scissors
- Small lockable boxes (5)
- Four-letter combination lock (1)
- Three-digit luggage locks (4)
- Four-digit luggage lock (1)
- iPad or other tablet (must be able to read a QR code)
- Transparency sized to match Patient Monitor screen

Printed Items from Appendices

- Set of blue Clue Cards [numbered 1-9]
- Set of yellow Exam Findings Cards [numbered 1-5]
- Set of vital sign symbols (for manikin and transparency)
- Temperature conversion chart (affixed to back of thermometer)
- BMI conversion chart
- AHA Blood Pressure chart
- BLS algorithm
- Patient chart cut into 4 puzzle pieces
- CDC Handwashing Charts
- Cards labelled "No Clue Here" for crash cart drawers not containing a clue

Manikin Set-Up

- Manikin lying supine on a bed clothed in street clothes
- Blanket covering manikin from chest to feet
- Heart shape pulse indicators on carotid artery and radial artery
- Diamond shape oxygen saturation indicator on finger
- QR code placed below belt line of pants (still hidden from view if shirt was raised)
- Blood pressure cuff on arm (Red square symbol affixed to the top of the pressure gauge)

All images and graphics are author-owned.

Overview of “Escape Room”

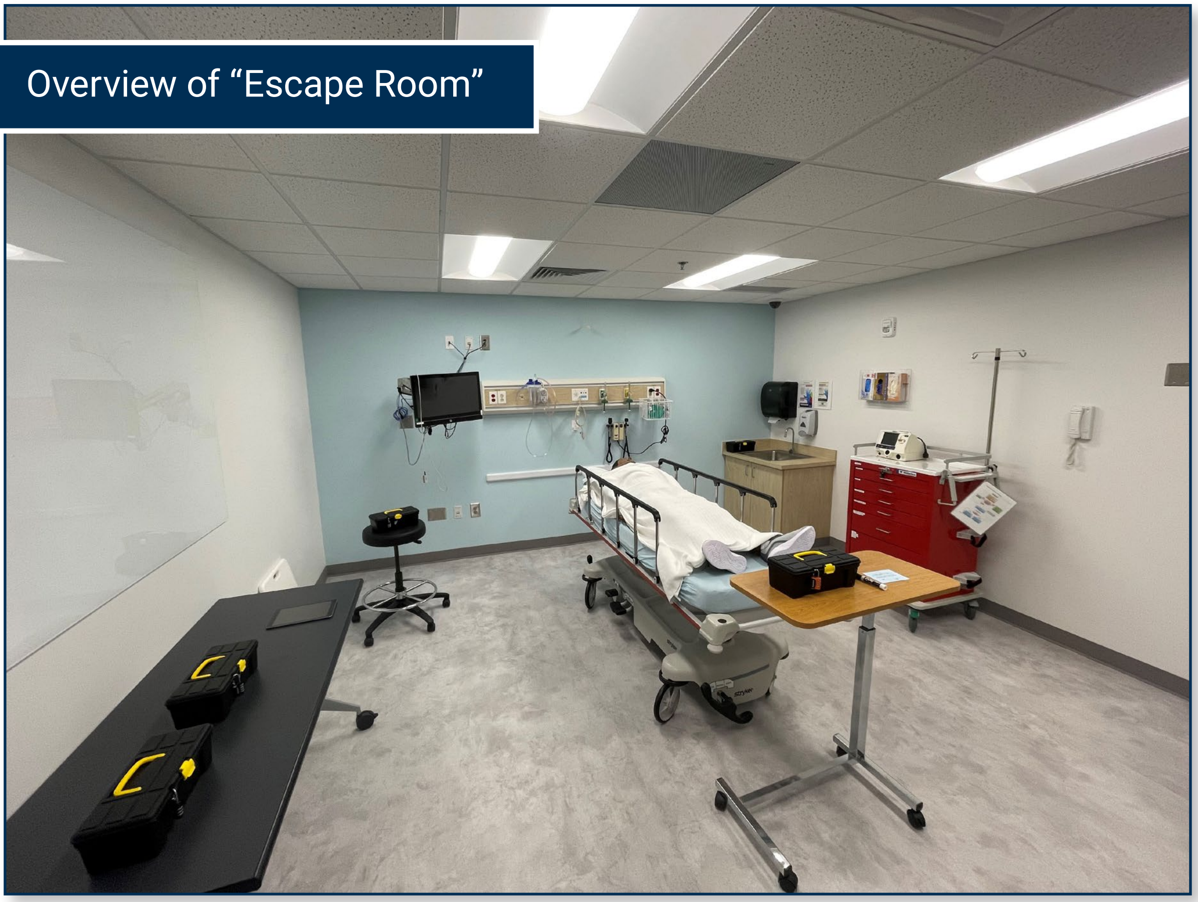

Box 1 contents

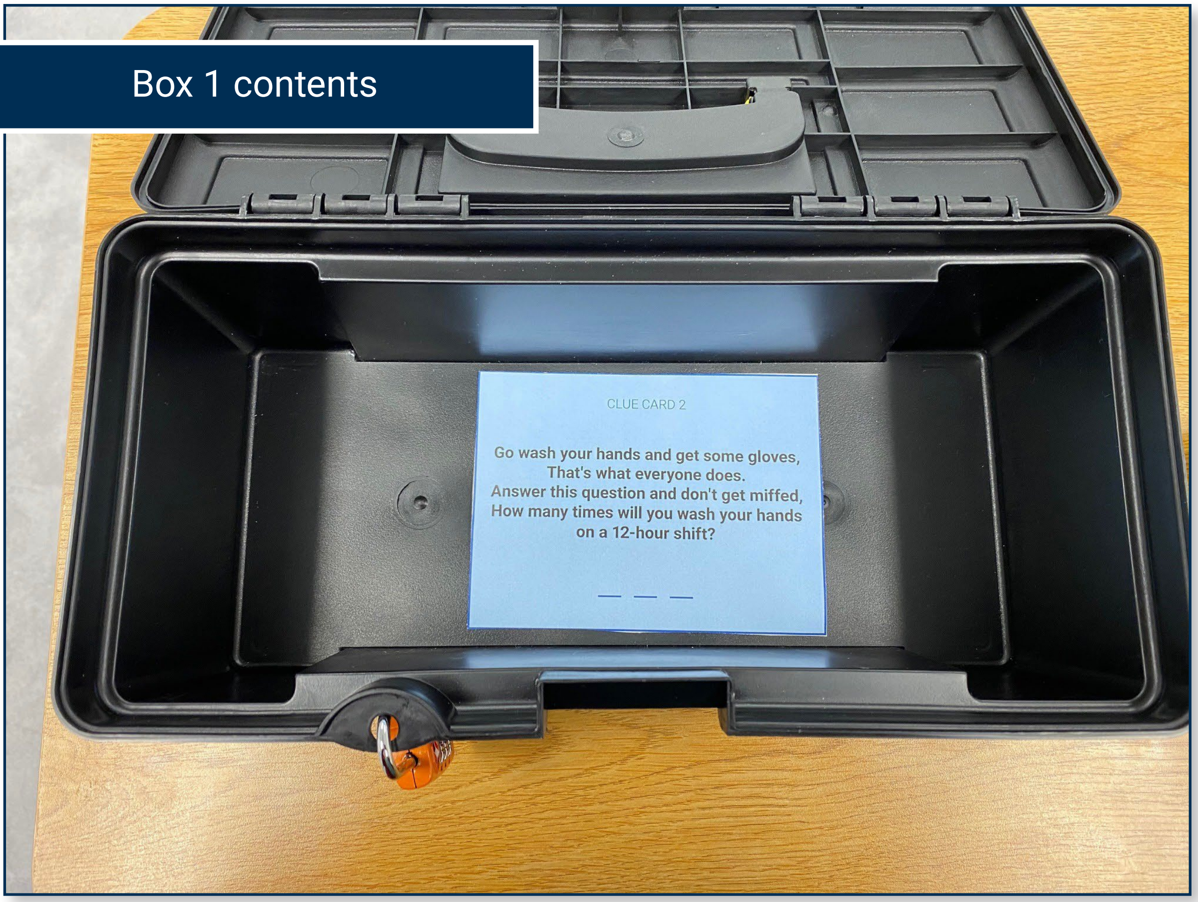

All images and graphics are author-owned.

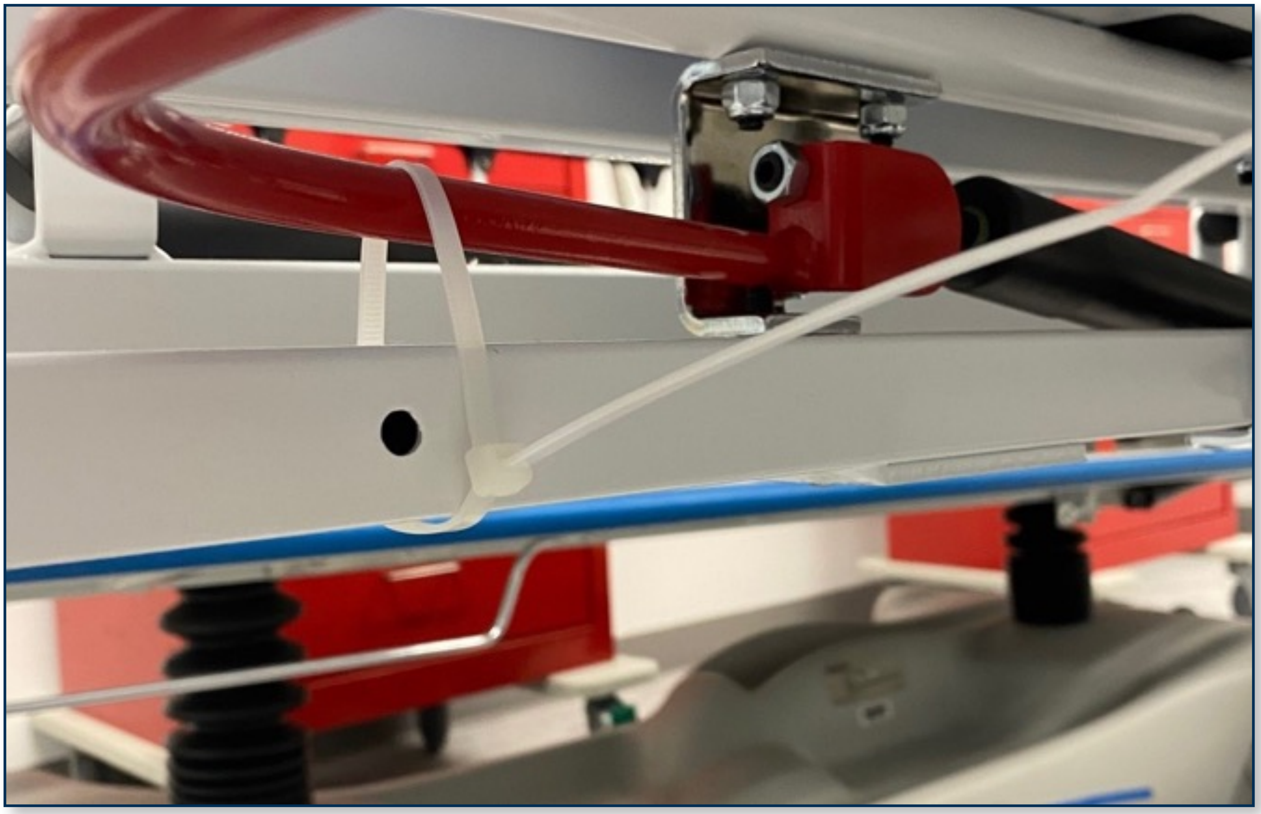

The bed adjustment lever is tied to the frame of the bed to prevent raising the head of the bed unless it is cut with the scissors provided in Box 2.

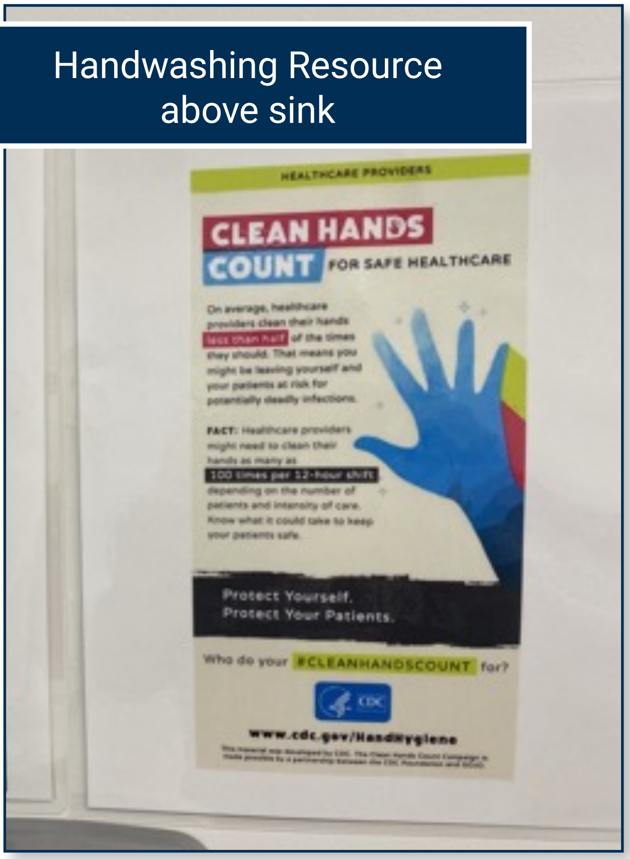

Handwashing Resource above sink

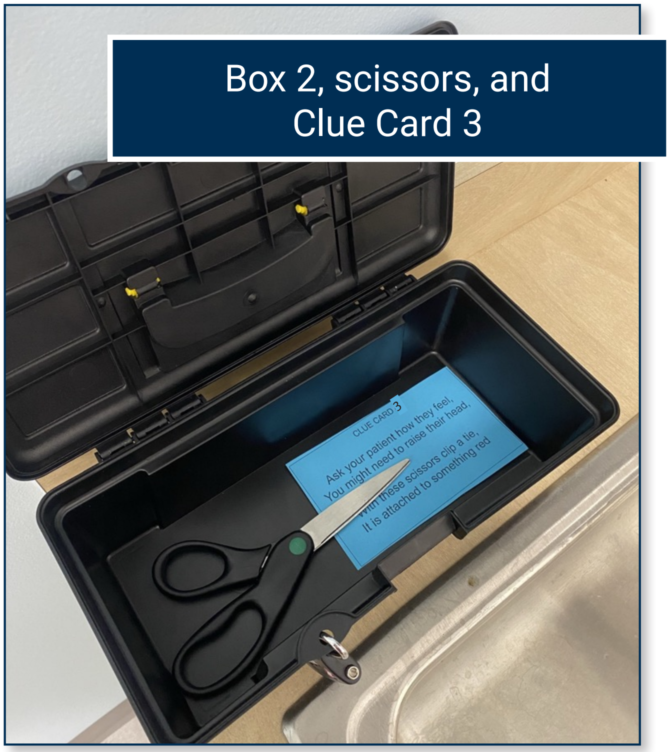

Box 2, scissors, and Clue Card 3

All images and graphics are author-owned.

Crash Cart with BLS & BP Charts

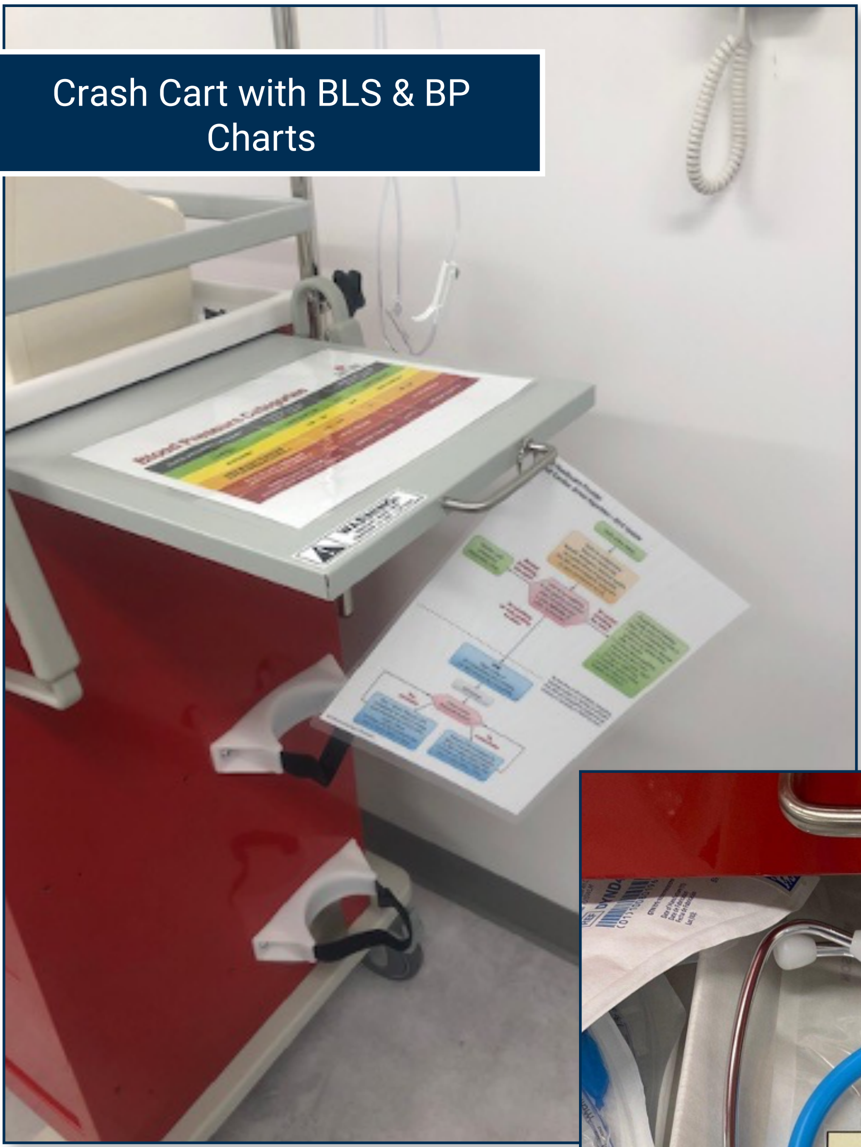

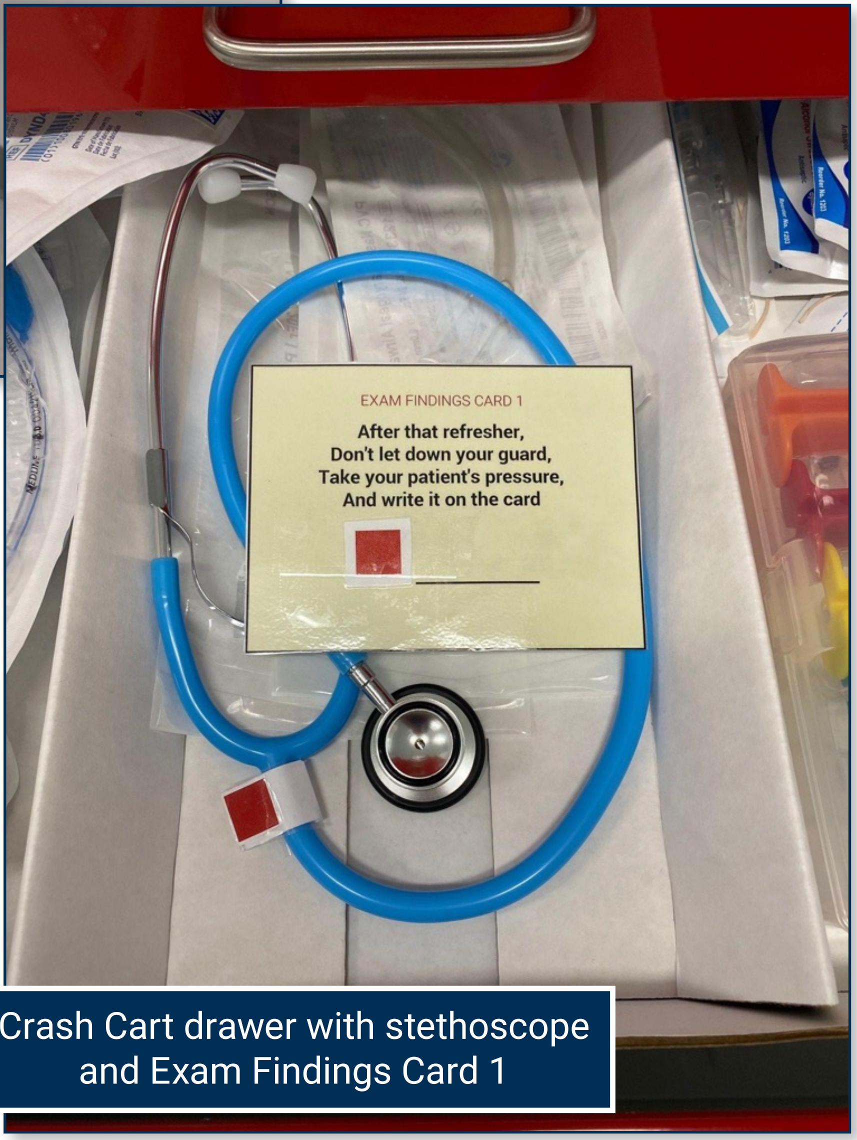

Crash Cart drawer with stethoscope and Exam Findings Card 1

All images and graphics are author-owned.

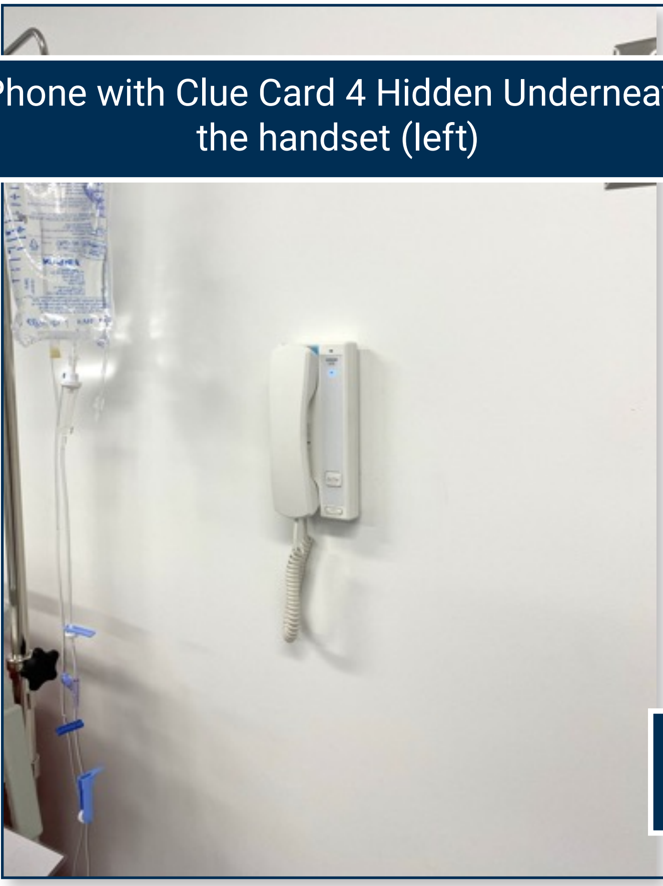

Phone with Clue Card 4 Hidden Underneath the handset (left)

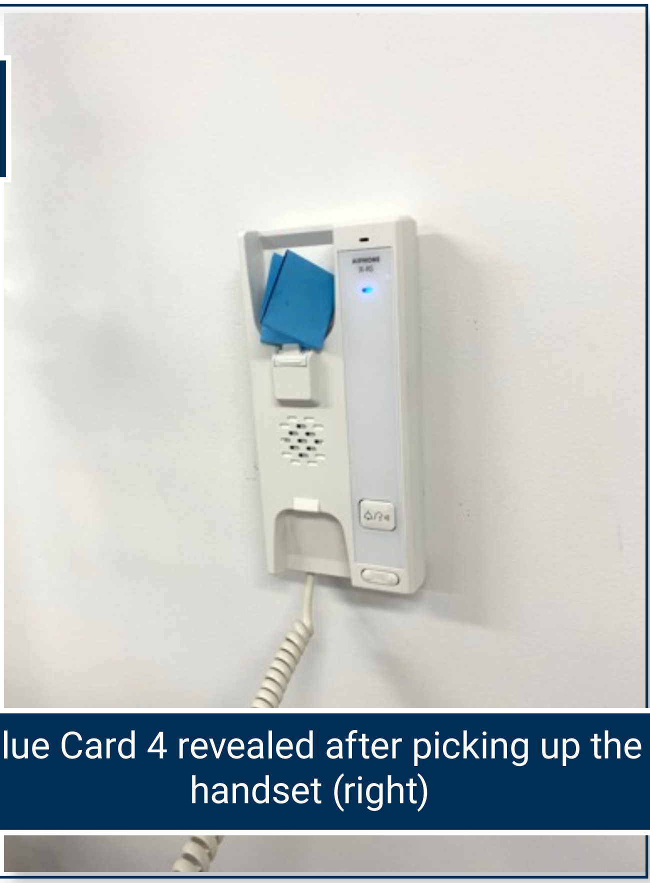

Clue Card 4 revealed after picking up the handset (right)

All images and graphics are author-owned.

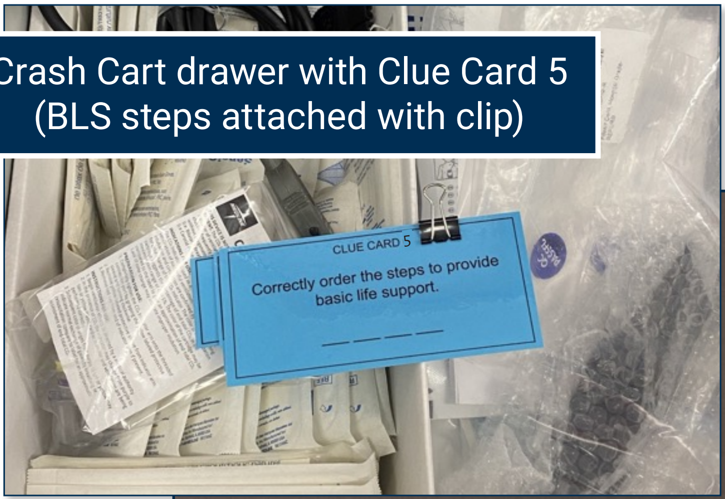

Crash Cart drawer with Clue Card 5 (BLS steps attached with clip)

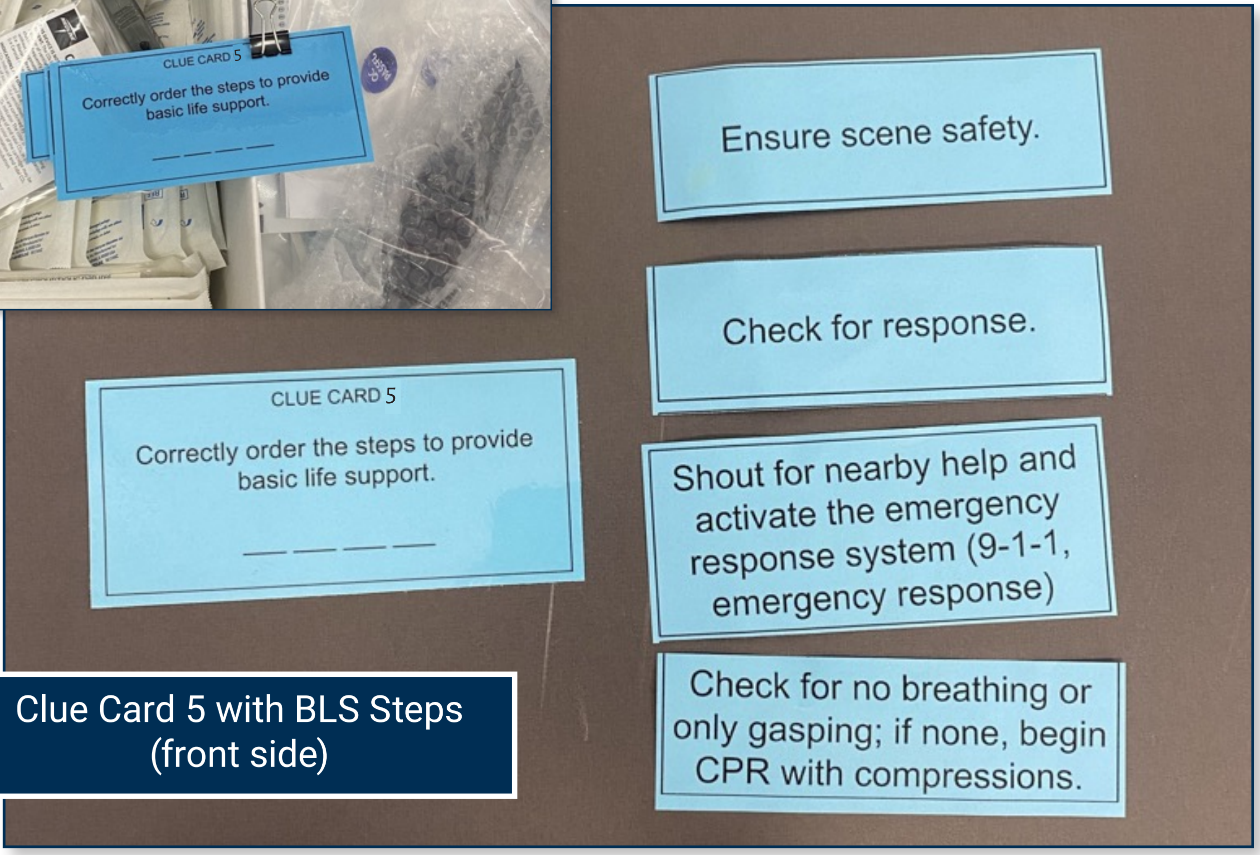

Clue Card 5 with BLS Steps (front side)

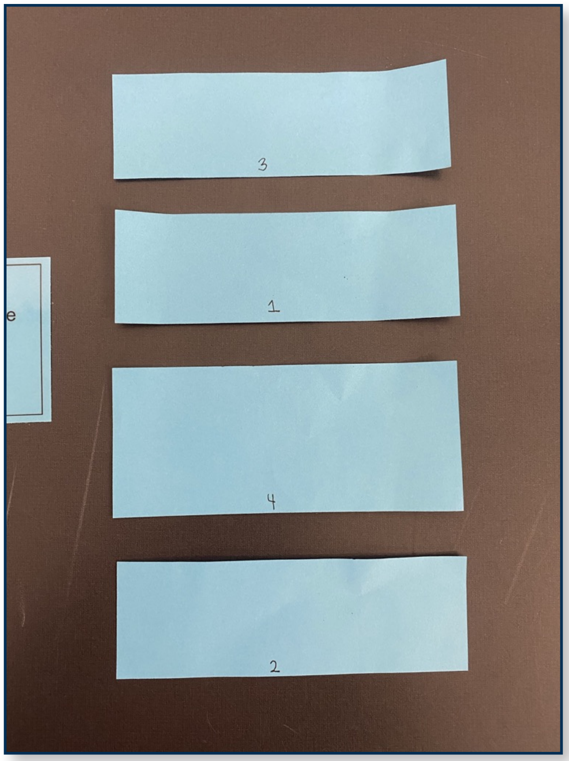

BLS Steps (Back side numbered to reveal the code 3142 when ordered correctly)

All images and graphics are author-owned.

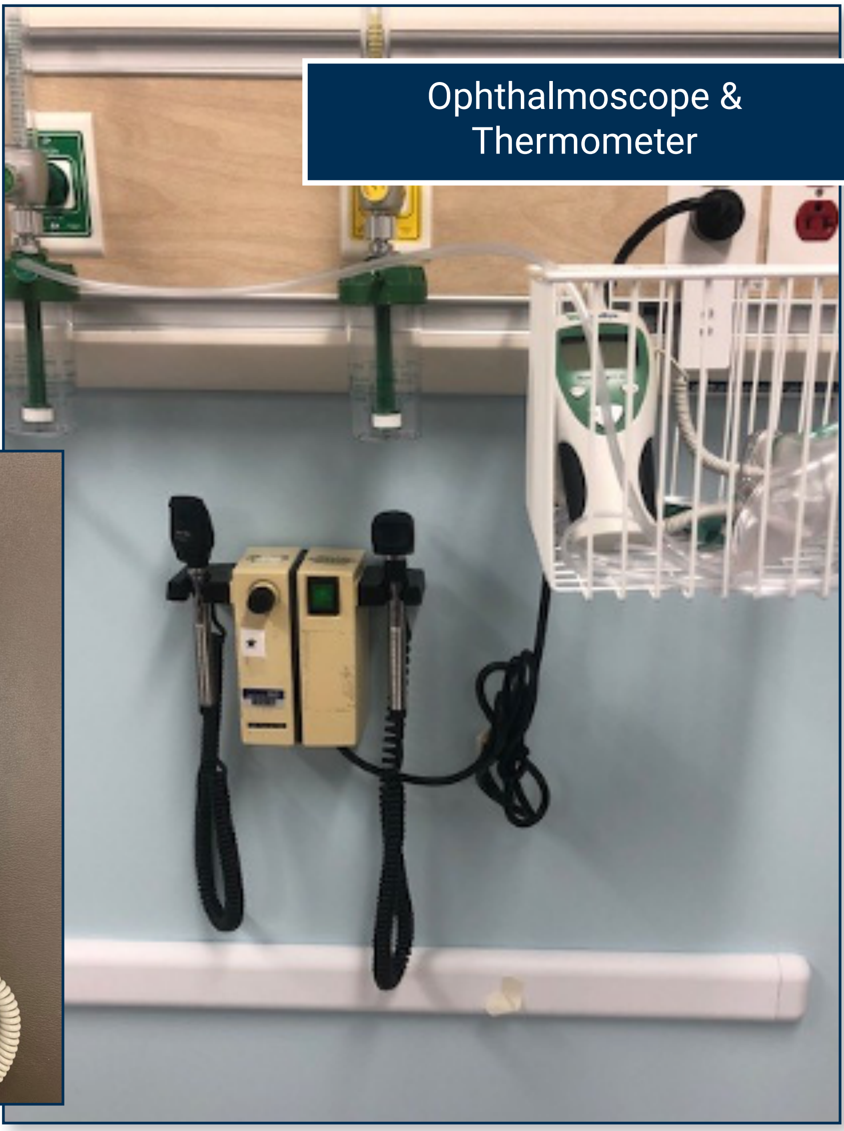

Ophthalmoscope & Thermometer

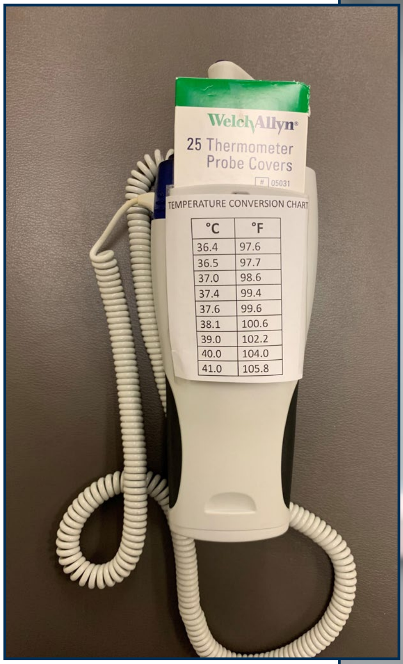

Back of thermometer

All images and graphics are author-owned.

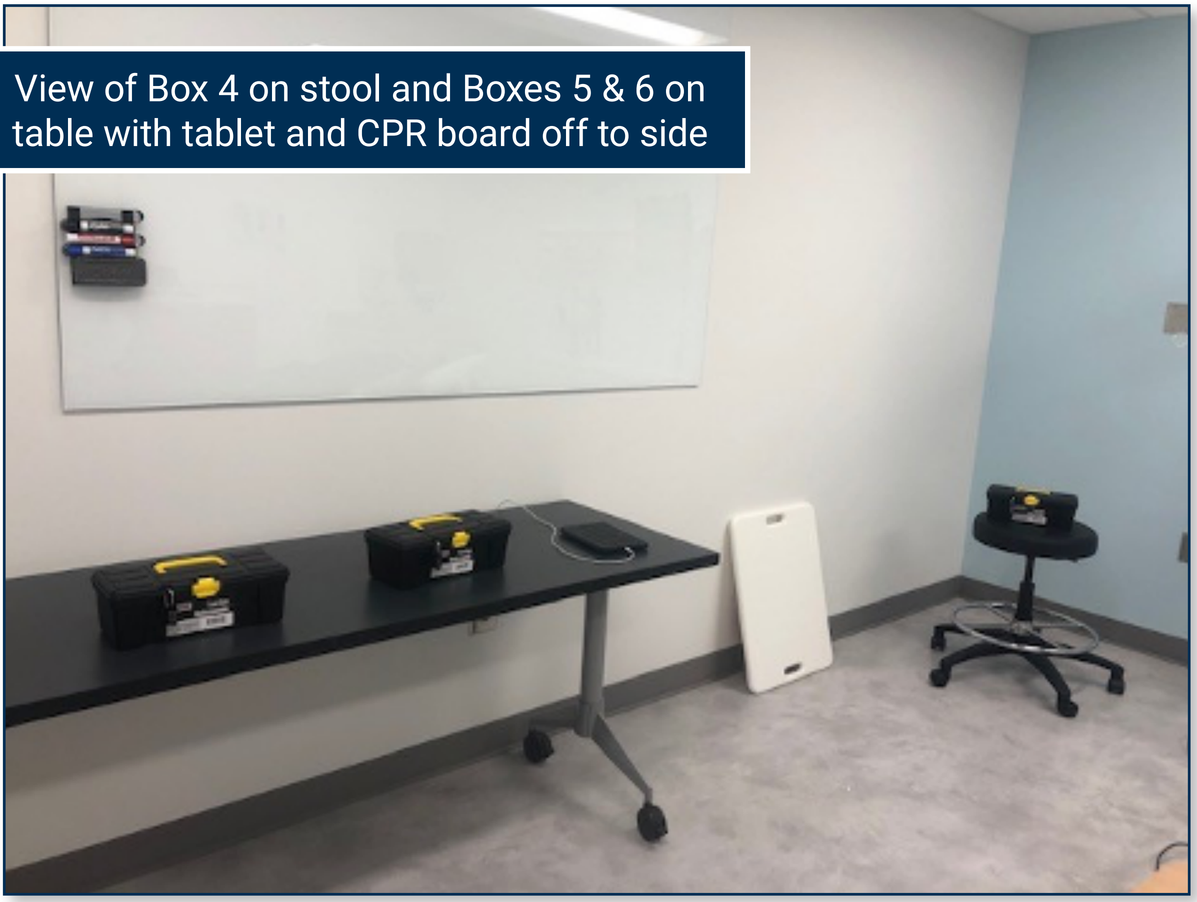

View of Box 4 on stool and Boxes 5 & 6 on table with tablet and CPR board off to side

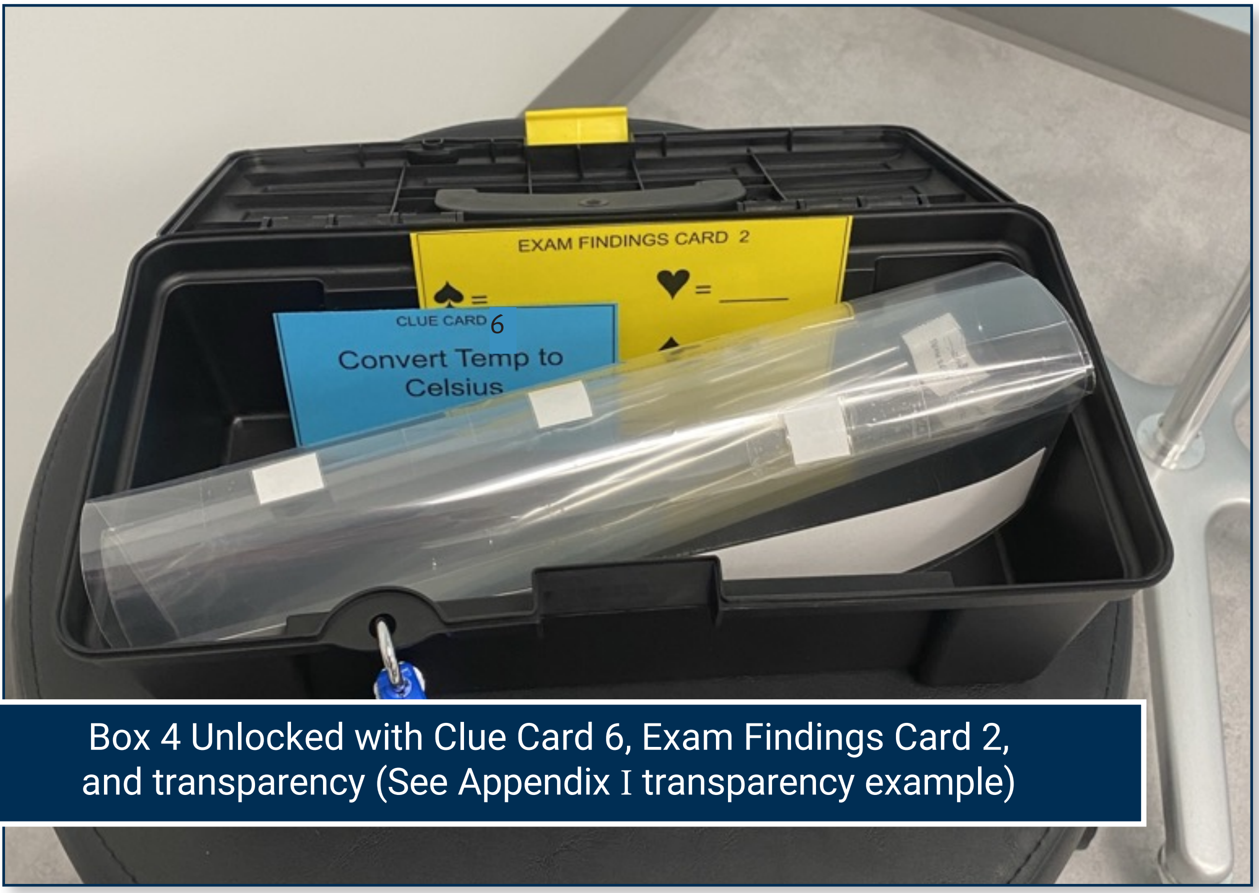

Box 4 Unlocked with Clue Card 6, Exam Findings Card 2, and transparency (See Appendix I transparency example)

All images and graphics are author-owned.

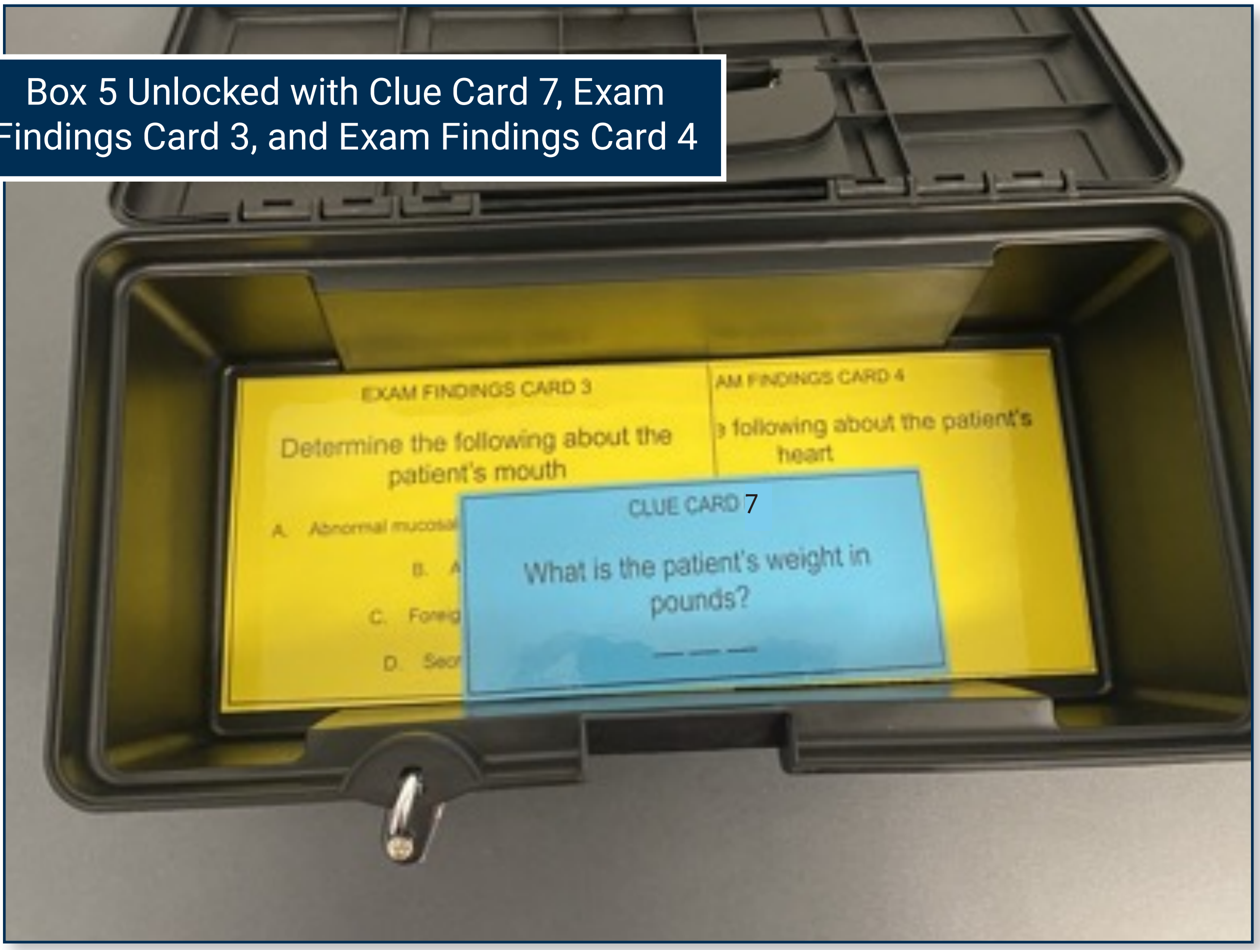

Box 5 Unlocked with Clue Card 7, Exam Findings Card 3, and Exam Findings Card 4

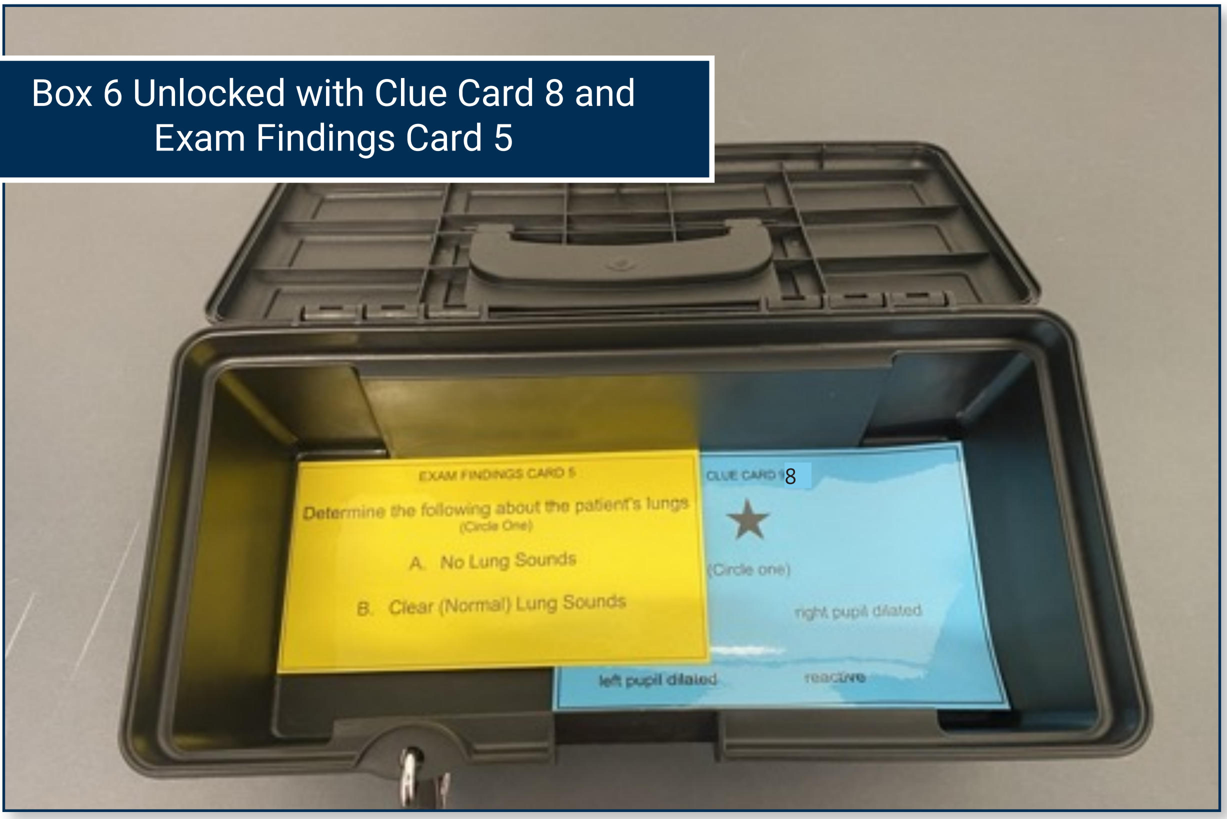

Box 6 Unlocked with Clue Card 8 and Exam Findings Card 5

All images and graphics are author-owned.

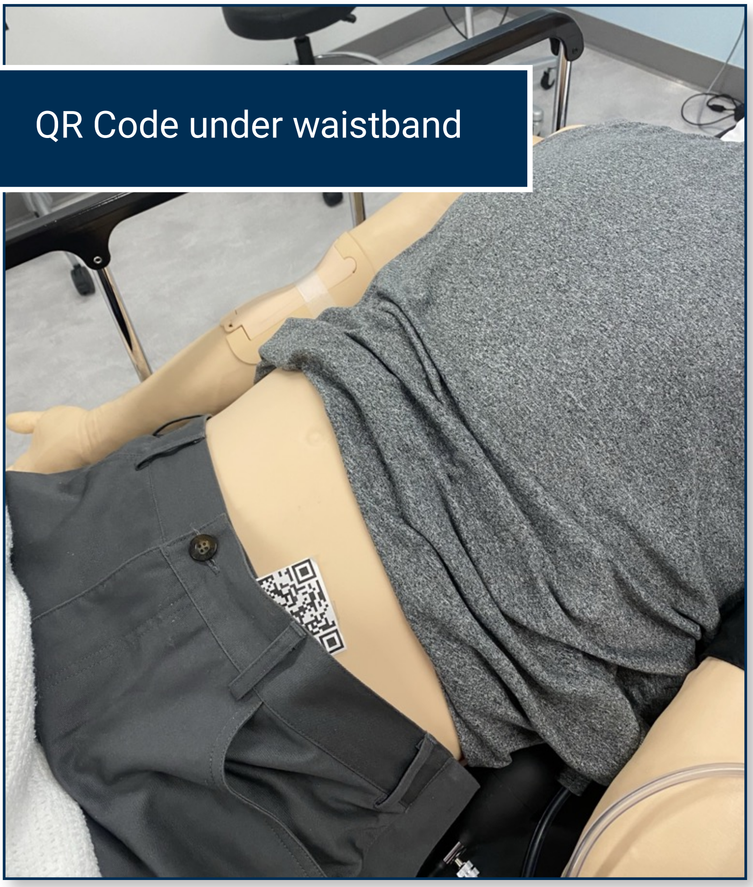
QR Code under waistband

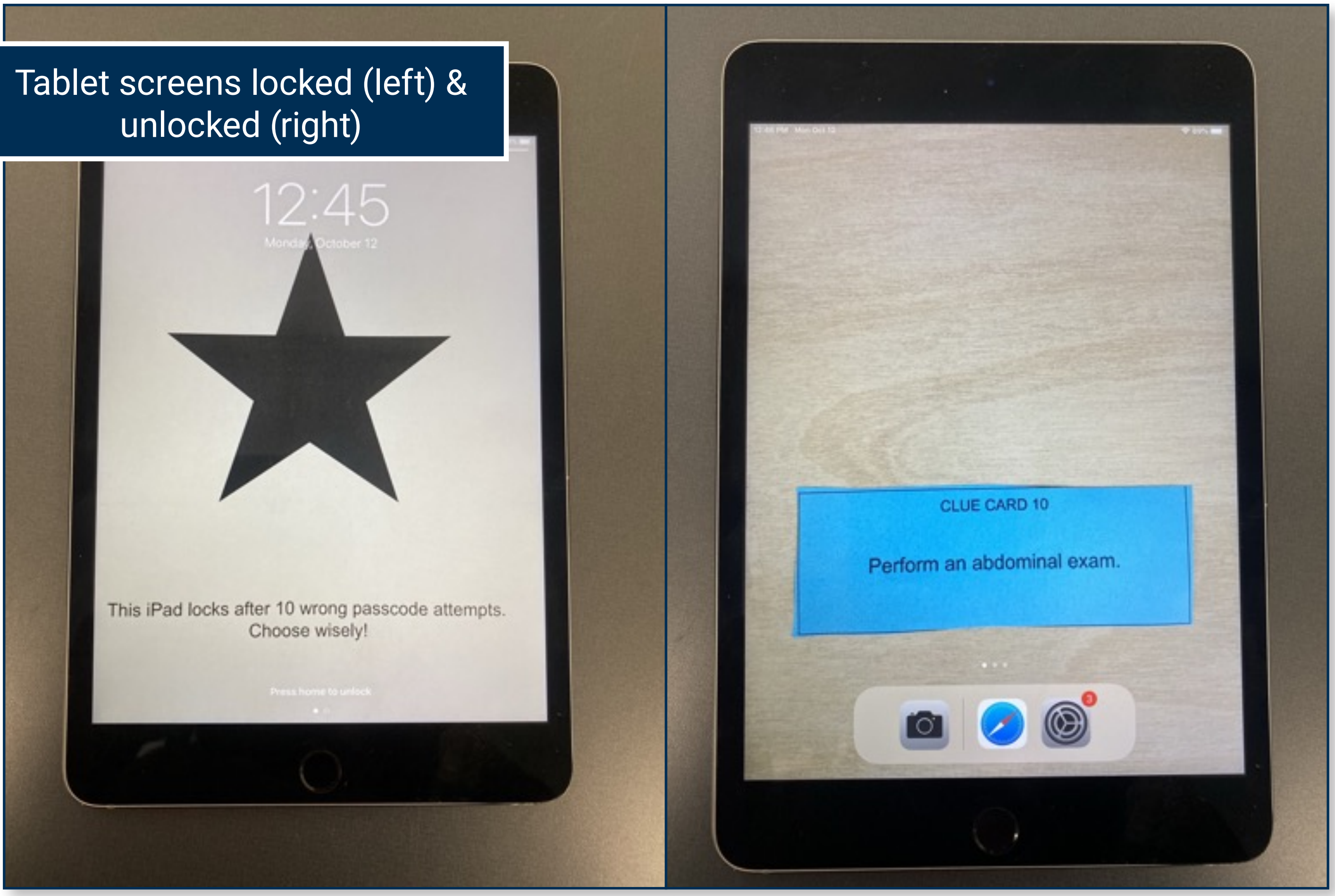

Tablet screens locked (left) & unlocked (right)

All images and graphics are author-owned.
