# Supplementary material for: An Escape Room to Orient Preclinical Medical Students to the Simulated Medical Environment
Source: MedEdPORTAL. 2022 Mar 25;18:11229. doi: 10.15766/mep_2374-8265.11229 (PMC8948100; doi:10.15766/mep_2374-8265.11229)
Supplement: Supplementary file 1 — Escape Room Simulation Guide.docxRoom Layout.pdfPatient Chart and Puzzle Template.pdfClue and Exam Findings Cards.pdfAdditional Room Resources.docxParticipant Prebriefing.pptxEscape Room Flow Chart and Codes.pdfExit Questionnaire.docxFaculty Instructions and Debriefing Guidelines.pdfCritical Actions Checklist.docxParticipant Evaluation.docxFollow-up Survey.docx [file mep_2374-8265.11229-s001.zip › D. Clue and Exam Findings Cards.pdf]

AUGUSTA UNIVERSITY
MEDICAL PARTNERSHIP
UNIVERSITY OF GEORGIA

Escape Room Activity
CLUE & EXAM FINDINGS CARDS

This appendix supplies the cards and symbol labels that are placed in specific locations within the escape room and on the manikin. Included are ten clue cards, five exam findings cards, nine symbols, one tablet wallpaper template, and two "no clue here" cards.

The blue clue cards are used to guide the participants through the escape room activity in a sequential fashion. The clue cards are hidden among four locked boxes and the locked crash cart. The cards are unlocked from their hidden locations in numerical order as the participants solve the puzzles and obtain the combinations to the locks. Clue card 1 is found first and clue card 9 is found last.

The yellow exam findings cards are also locked among the four boxes and crash cart. They are found in numbered order. The purpose of the exam findings cards is to prompt participants to perform an exam based on the symbol or question that is presented on the card and write in their findings on the cards. For example, the heart symbol is shown next to a blank on exam findings card 2. The participants discover that the manikin has the same heart shape on the radial pulse. The participants check the pulse and write the finding in the blank on the exam findings card.

Exam Findings Cards Symbols Key:

♠ = Respiratory Rate
♥ = Heart Rate
♣ = Temperature
♦ = SpO_2
■ = Press the red square on the transparency for automated blood pressure
← Press here = Directly to the right of the red square on the transparency so participants recognize the red square is a button that they needed to press

After printing the document, cut and arrange as described (see small print notations above or below each item) in the room and on the manikin.

The lock screen card that reads "This iPad locks after 10 wrong attempts. Choose wisely!" with the star symbol should be used as the background of the tablet lock screen. Save and crop the image provided (or take a photograph with the tablet camera) and set as the tablet "wallpaper". When the participants turn on the tablet screen, the photo of the card will appear. The tablet is programmed with the Alphanumeric Code function that requires the tablet to be unlocked with a word. In this case, the word is "reactive", as found from the answer to clue card 8.

The cards that read "No Clue in Here" were placed in the drawers of the crash cart that didn't contain any clue cards, exam findings cards or anything else that was part of the activity. The cards were necessary in order to keep the groups from spending too much time looking through drawers without clues.

The QR code is the last piece that the participants have to find. The QR code can be cut out and placed on the manikin's lower abdomen, below the clothing waistband, to be found when the participants perform the abdominal exam.

Photos in Appendix B, Room Layout, show the placement of all pieces within the room. Appendix G is a quick reference sheet that describes the location of each clue/exam findings card.

Extra symbols are included in this appendix.

All images and graphics are author-owned.

CLUE CARD 1

There was a chart on the door,
But now it's broken into ***four***.
It's a ***puzzle***, I presume.
Find the ***pieces*** in the room.

“W _ _ _ _ _ _ _
_ _ _ _ S !”

↑ (On table at the foot of the bed) [blue]

Cut all clue cards out along the blue lines

CLUE CARD 2

Go wash your hands and get some gloves,
That's what everyone does.
Answer this question and don't get miffed,
How many times will you wash your hands on a 12-hour shift?

_ _ _

↑ (In Box #1) [blue]

↓ (In Box #2) [blue]

↓ (Taped over phone earpiece) [blue]

CLUE CARD 3

Ask your patient how they feel,
You might need to raise their head,

With these scissors clip a tie,
It is attached to something RED

CLUE CARD 4

What is the highest systolic blood pressure value that is considered normal?

_ _ _

All images and graphics are author-owned.

CLUE CARD 5

Correctly order the steps to provide basic life support.

___ ___ ___ ___

CLUE CARD 6

Convert temperature to Celsius

___ ___ . ___

↑ (Place in the bottom drawer of the crashcart (Box #3) with the 4 BLS cards on the last page) [blue]

↑ (Box #4) [blue]

↓ (Box #5)

↓ (Box #6)

CLUE CARD 7

What is the patient's weight in pounds?

___ ___ ___

CLUE CARD 8

(Circle One)

non-reactive **left pupil dilated**

reactive **right pupil dilated**

All images and graphics are author-owned.

↓ (Take a photo of this card and set as the tablet HOME screen.)

CLUE CARD 9

Perform an abdominal exam.

(Cut out and place QR code underneath waistband of pants/shorts of manikin - must be low enough to not be seen unless clothing is moved)

All images and graphics are author-owned.

Ensure scene safety.

Shout for nearby help and activate the emergency response system

(9-1-1, emergency response).

↑ (Write the number 3 on the back of this card)
↓ (Write the number 1 on the back of this card)

(Cut out and place all 4 of these cards in the bottom drawer of crash cart with Clue Card #5.)

↑ (Write the number 4 on the back of this card)
↓ (Write the number 2 on the back of this card)

Check for response.

Check for no breathing or only gasping;
if none, begin CPR with compressions.

All images and graphics are author-owned.

EXAM FINDINGS CARD 1

**After that refresher,
Don't let down your guard,
Take your patient's pressure,
And write it on the card.**

■ = ________

↑ (3rd drawer of crash cart) [yellow]

↓ (Box #5) [yellow]

Cut all exam findings cards out along the black lines

EXAM FINDINGS CARD 2

♠ = ________ ♥ = ________

♥ = ________ ♦ = ________

■ = ________

↑ (In Box #4) [yellow]

↓ (In Box #5) [yellow]

EXAM FINDINGS CARD 3

Determine the following about the patient's mouth:

- Abnormal muscosal or skin discoloration present
- Airway clear
- Foreign object present
- Secretions present

EXAM FINDINGS CARD 4

Determine the following about the patient's heart: *(circle one)*

No Heart Sounds

Regular Rhythm

Irregular Rhythm

All images and graphics are author-owned.

EXAM FINDINGS CARD 5

Determine the following about the patient's lungs: *(circle one)*

No Lung Sounds

Clear (Normal) Lung Sounds

↑ (In Box#6) [yellow]

Symbols below are taped to transparency to indicate corresponding patient monitor vitals:

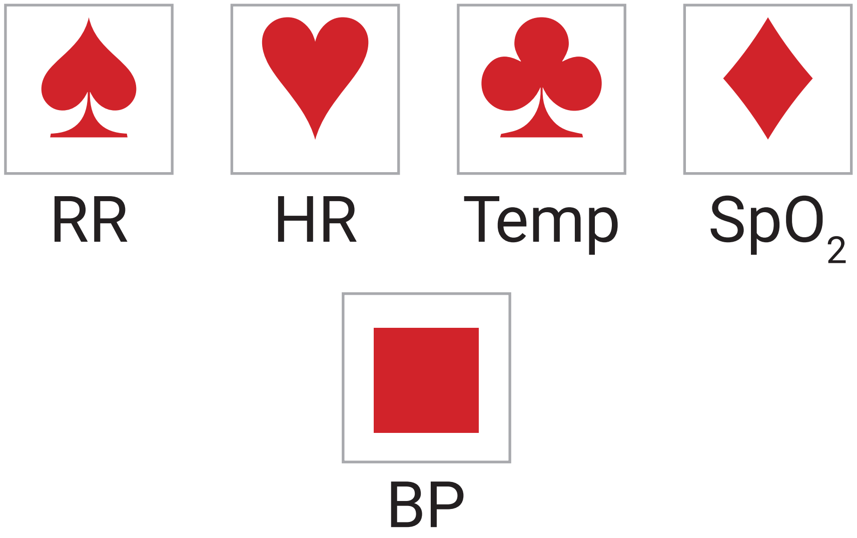

All images and graphics are author-owned.

Extra Symbols

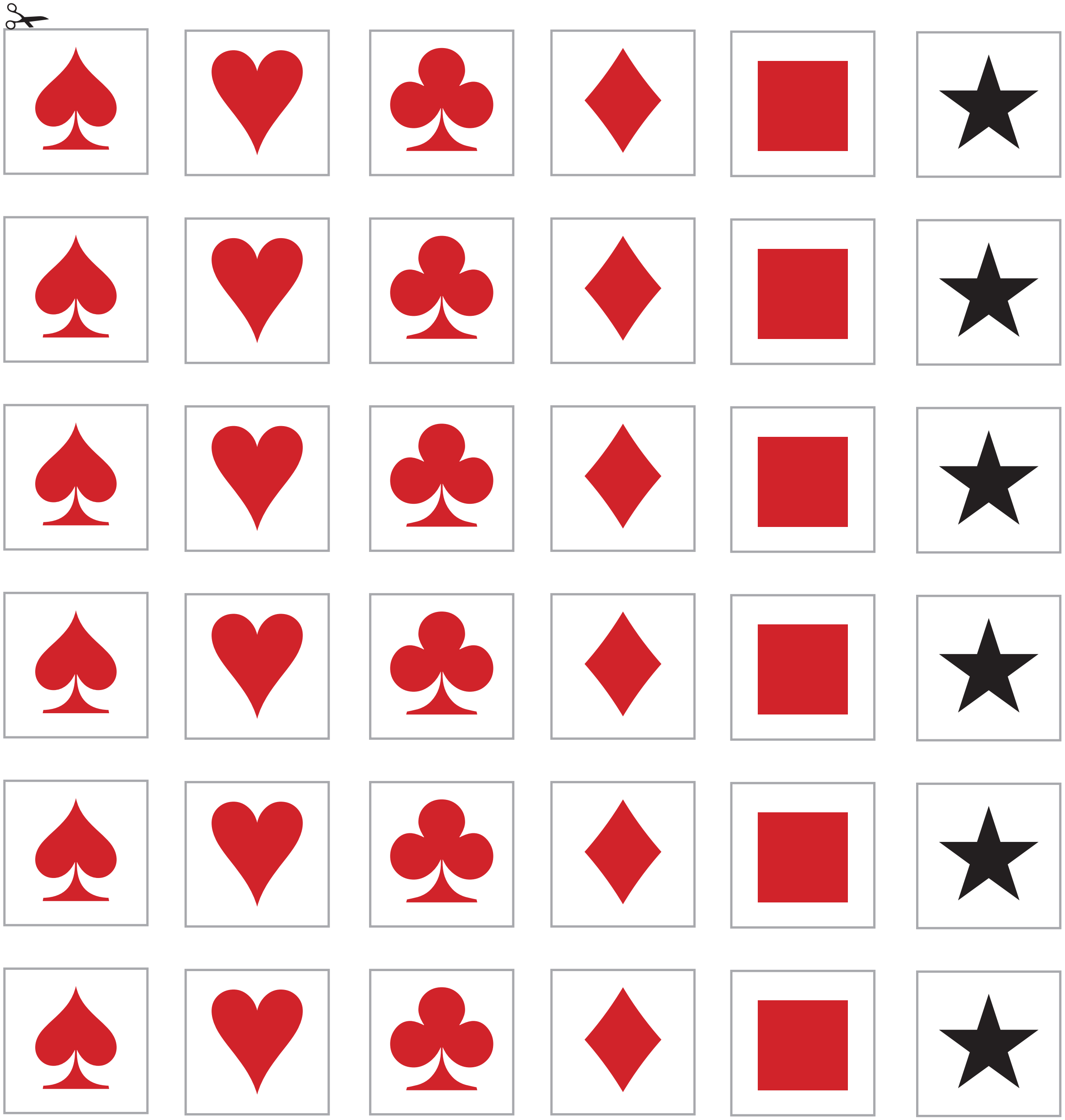

← Press here

← Press here

← Press here

← Press here

All images and graphics are author-owned.

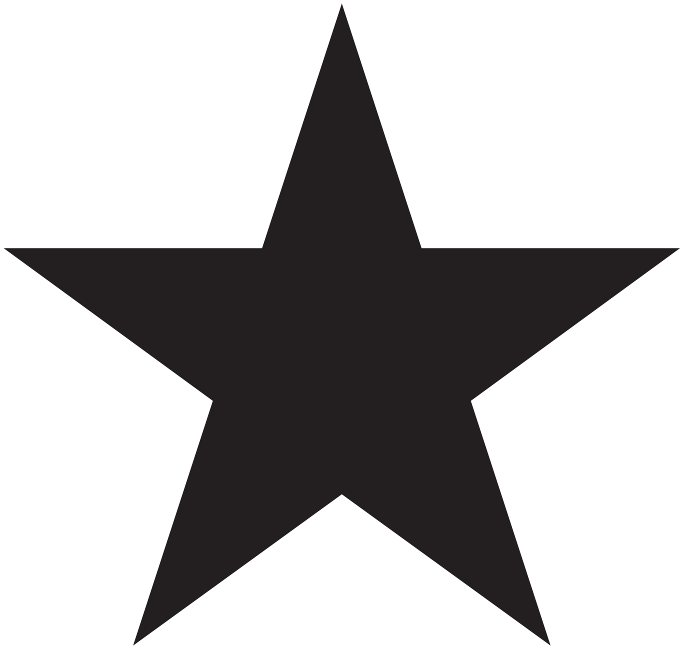

This iPad locks after 10 wrong passcode attempts.

Choose wisely!

Take a photo of this card and set at the tablet LOCK screen.

No Clue in Here

(Place in any crash cart drawer without a clue card.)

No Clue in Here

All images and graphics are author-owned.
