# Supplementary material for: An Escape Room to Orient Preclinical Medical Students to the Simulated Medical Environment
Source: MedEdPORTAL. 2022 Mar 25;18:11229. doi: 10.15766/mep_2374-8265.11229 (PMC8948100; doi:10.15766/mep_2374-8265.11229)
Supplement: Supplementary file 1 — Escape Room Simulation Guide.docxRoom Layout.pdfPatient Chart and Puzzle Template.pdfClue and Exam Findings Cards.pdfAdditional Room Resources.docxParticipant Prebriefing.pptxEscape Room Flow Chart and Codes.pdfExit Questionnaire.docxFaculty Instructions and Debriefing Guidelines.pdfCritical Actions Checklist.docxParticipant Evaluation.docxFollow-up Survey.docx [file mep_2374-8265.11229-s001.zip › I. Faculty Instructions and Debriefing Guidelines.pdf]

AUGUSTA UNIVERSITY
MEDICAL PARTNERSHIP
UNIVERSITY OF GEORGIA

Faculty Instructions & Debriefing GUIDELINES

The Escape Room is a hands-on activity to orient preclinical medical students to the simulation bay, some basic equipment in the room, and features of the patient manikin.

The learners are asked to complete a follow up visit for a patient in a primary care clinic, including performing and documenting a basic physical exam of the patient before they can escape the room. There are 12 tasks that need to be completed to escape. Tasks are identified with a puzzle to solve and/or a clue provided in the room. The puzzles and clues are revealed in sequential order, but are not required to be completed in a particular order.

The learners will attend a 10-minute pre-briefing describing the scenario and the activity rules, and then will be 'locked' in the room. They will have 40 minutes to complete the tasks and escape the room. Faculty will be operating the manikin, providing hints to the learners if needed, and documenting completion of the tasks on the Critical Actions Checklist. At 30 minutes, the faculty operator will announce "10 Minutes remaining". Once 40 minutes has elapsed, the operator will announce "The session has ended". Immediately following the session, the faculty operator will meet with the learners to debrief the activity. There will be approximately 40 minutes available for debriefing. The debriefing may take place in the simulation bay to better address questions on equipment or manikin function.

The learners are NOT allowed to bring any resources (phones, tablets, books) into the escape room for this activity.

Try not to give the answers to clues or direct learners to resource locations, if possible. Instead ask them to rethink their answer or advise that they search further for one of the provided room resources if they haven't found it yet. However, the goal is for all groups to complete all tasks and escape the room. Use your own discretion to assist them if they are stuck and in danger of not completing the room. You may give clues using the manikin speaker or using the room phone. Try to stay in the role of a patient if using the manikin speaker.

Escape Room Debriefing Points

Use the Critical Actions Checklist to guide the debriefing. During debriefing, please help the learners to reflect on their experience, review the room layout, and discuss use of equipment and manikin features. Please use the PEARLS Healthcare Debriefing Tool to help guide you.

Suggestions for Debriefing TOPICS or QUESTIONS or POINTS:

1. **It can be startling when the manikin talks to you. How did interacting with the patient manikin feel as compared to a person?**
2. **The activity requires that you become aware of and use resources found in the room (phone, crash cart items, BLS algorithm, BMI chart, Temp conversion chart). How might these types of resources (medication dosing charts, clinical decision rules, etc.) be useful in caring for acutely ill patients, and why?**
3. **It looked like you had trouble feeling the pulses/auscultating the lungs/seeing in the mouth/etc. What did you experience? The manikins have many limitations, and we realize that they can be difficult to**

examine. In future simulation activities, you will have a clinician playing a nurse in the room with you. You can ask the nurse to confirm your physical exam findings if you are unsure of what you are seeing/hearing/feeling, etc., and they will provide you with the correct information while staying in the role of the nurse.

Some key points about the room and manikin:

- ❑ Importance of hand hygiene and use of gloves.
- ❑ The crash carts have BLS and ACLS guidelines, supplies for peripheral IV placement, airway management equipment, simulated medications, a defibrillator and IV fluids. The nurse will help them find and use supplies in future simulation activities. They should not use equipment that they have not been trained to use.
- ❑ The manikin BP can be taken manually **on the left arm**, or it can be taken as an automated function of the monitor.
- ❑ Vital Signs are displayed on the monitor-"HR" shows heart rate detected by ECG leads. On some manikins, RR is linked to ECG leads and will be visible when they are activated. "Pulse" shows arterial pulsation rate detected by the SPO2 finger probe. The values for HR and Pulse should be the same. Some manikins have bilateral carotid, radial, femoral, posterior tibial, and dorsalis pedis pulses.
- ❑ The manikin pupils are reactive. A pupillary exam can aid in differential diagnoses, as pupils that are dilated, pinpoint, unequal, or unreactive can all signify an underlying disorder. If they thought the pupils were unreactive, it may be because the room was too bright-they can dim the lights (and should for real patients) for a better exam.
- ❑ The manikin mouth can show cyanosis, erythema, tongue swelling, and can have secretions. May hear audible airway sounds such as stridor in some manikins (speaker in neck).
- ❑ Speakers in the chest over the typical areas of auscultation for the lungs and heart can play various sounds to replicate wheezing, crackles, heart murmurs, etc. The manikin compressor, which is responsible for causing the visible chest rise and palpable pulses, can make noises obscuring the speakers. Operator can pause the compressor to make sounds more clear.
- ❑ Speakers in the abdomen can play various intestinal sounds. The abdomen **cannot** display guarding, distention.

Patient Monitor Vital Signs key	
♥	Heart rate
♠	Respiratory rate
♣	Temperature
♦	Oxygen saturation
■	Blood Pressure

Transparency example images on following pages.

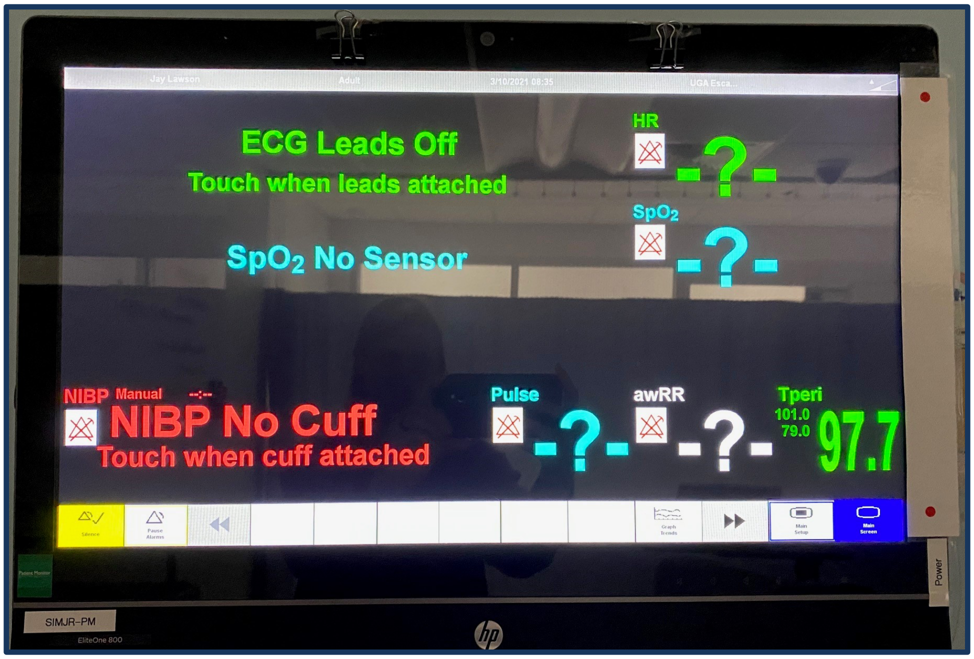

Monitor *WITHOUT* Transparency

Transparency Placement Guide (with two red dots) placed along the right side of the monitor.

↵

Monitor *WITH* Transparency

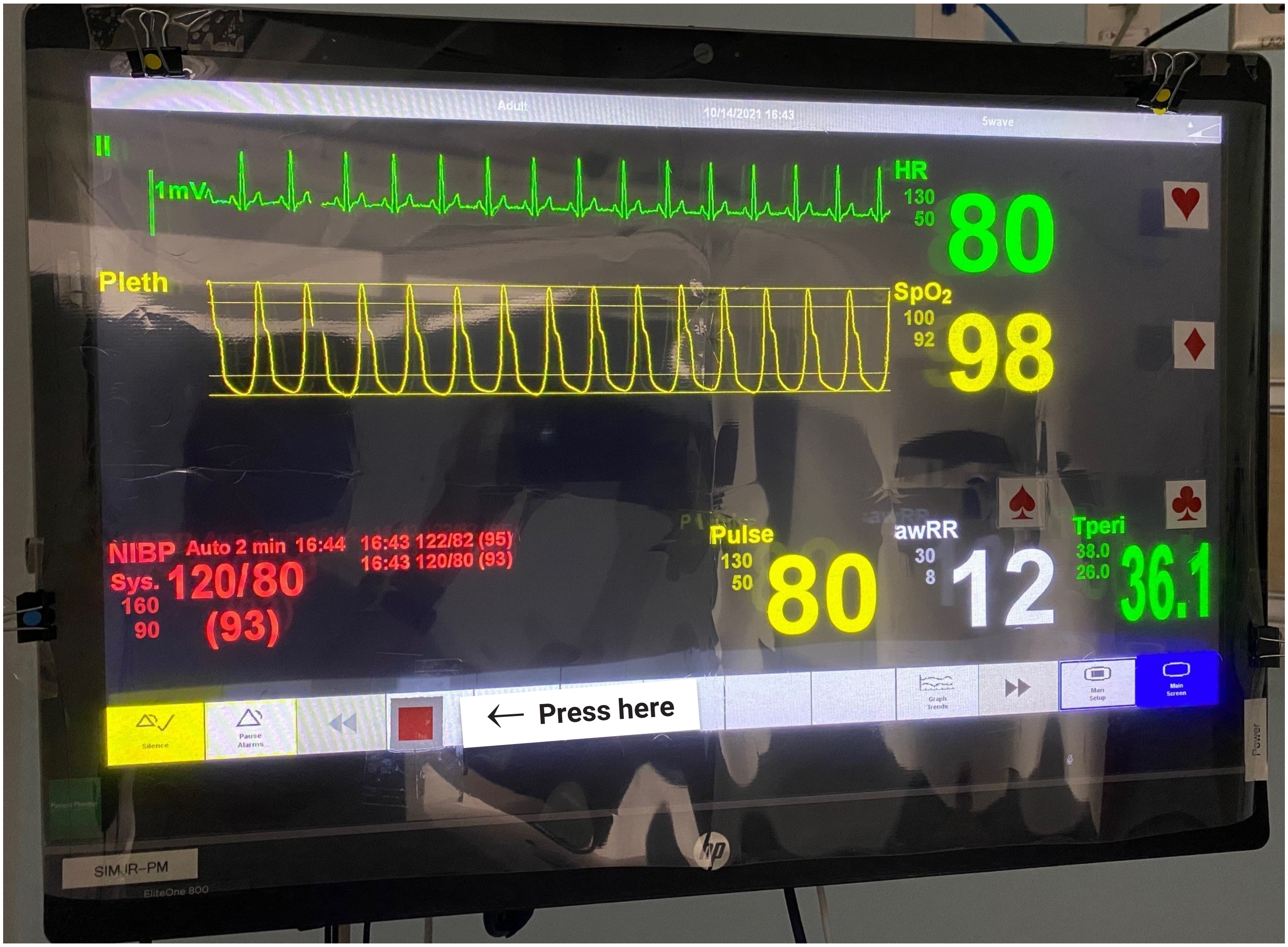

All images are author-owned.
