# Supplementary material for: An Escape Room to Orient Preclinical Medical Students to the Simulated Medical Environment
Source: MedEdPORTAL. 2022 Mar 25;18:11229. doi: 10.15766/mep_2374-8265.11229 (PMC8948100; doi:10.15766/mep_2374-8265.11229)
Supplement: Supplementary file 1 — Escape Room Simulation Guide.docxRoom Layout.pdfPatient Chart and Puzzle Template.pdfClue and Exam Findings Cards.pdfAdditional Room Resources.docxParticipant Prebriefing.pptxEscape Room Flow Chart and Codes.pdfExit Questionnaire.docxFaculty Instructions and Debriefing Guidelines.pdfCritical Actions Checklist.docxParticipant Evaluation.docxFollow-up Survey.docx [file mep_2374-8265.11229-s001.zip › F. Participant Prebriefing.pptx]

## Slide 1
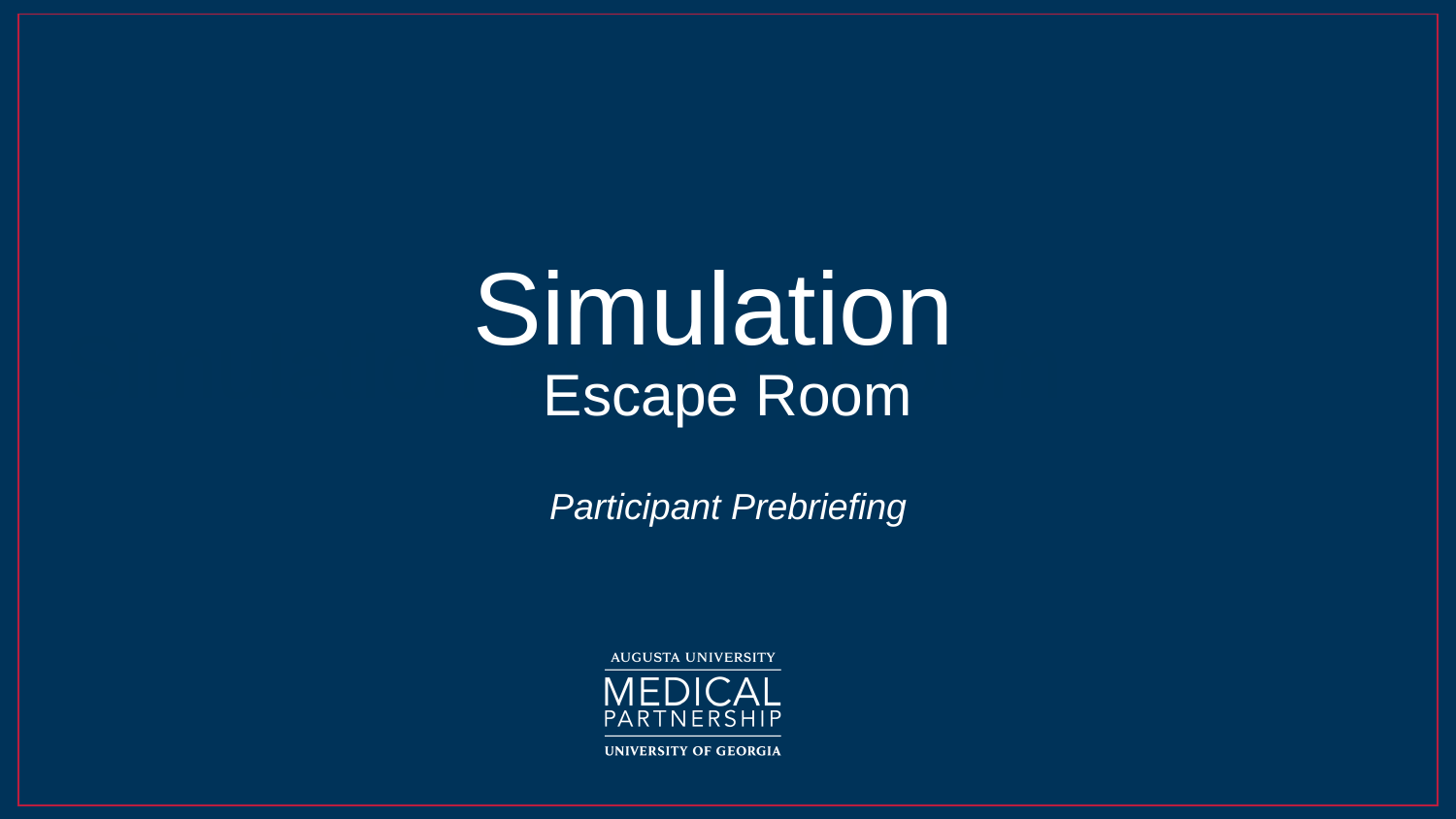

Simulation
Simulation Escape Room
Escape Room
Participant Prebriefing

## Slide 2
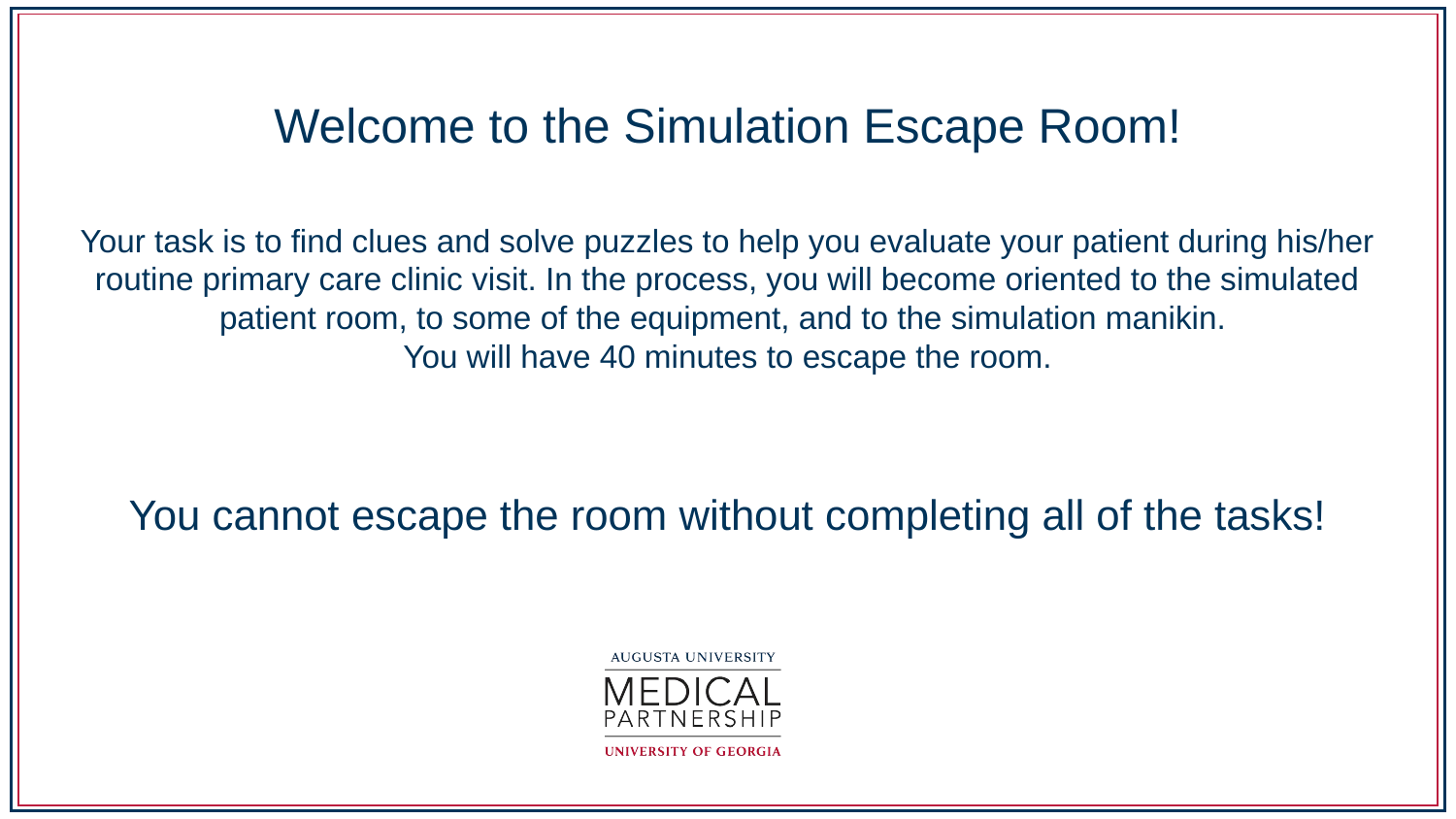

Welcome to the Simulation Escape Room!
Your task is to find clues and solve puzzles to help you evaluate your patient during his/her routine primary care clinic visit. In the process, you will become oriented to the simulated patient room, to some of the equipment, and to the simulation manikin.
You will have 40 minutes to escape the room.
You cannot escape the room without completing all of the tasks!

## Slide 3
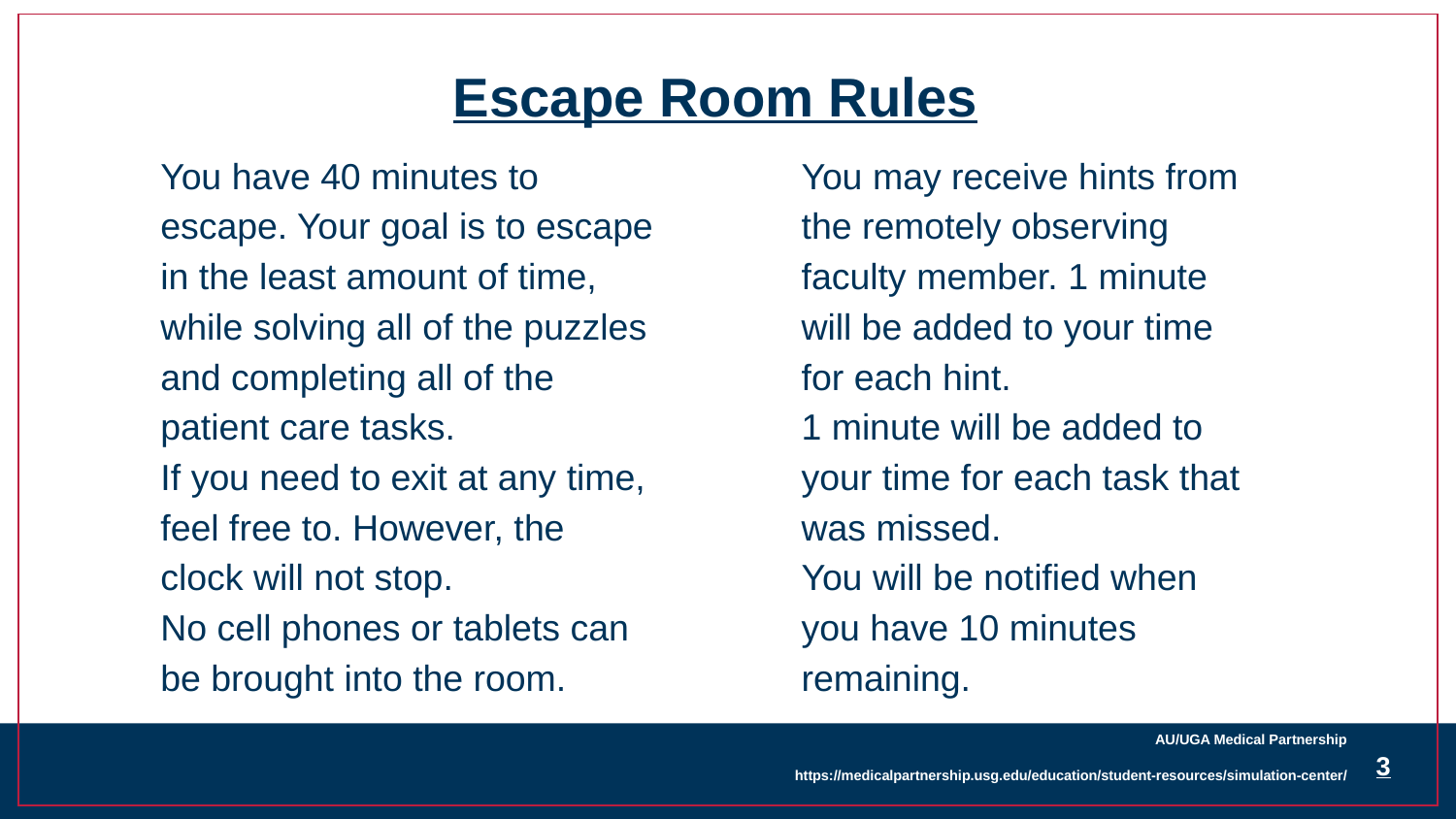

Escape Room Rules
You have 40 minutes to escape. Your goal is to escape in the least amount of time, while solving all of the puzzles and completing all of the patient care tasks.
If you need to exit at any time, feel free to. However, the clock will not stop.
No cell phones or tablets can be brought into the room.
You may receive hints from the remotely observing faculty member. 1 minute will be added to your time for each hint.
1 minute will be added to your time for each task that was missed.
You will be notified when you have 10 minutes remaining.
AU/UGA Medical Partnership
https://medicalpartnership.usg.edu/education/student-resources/simulation-center/

## Slide 4
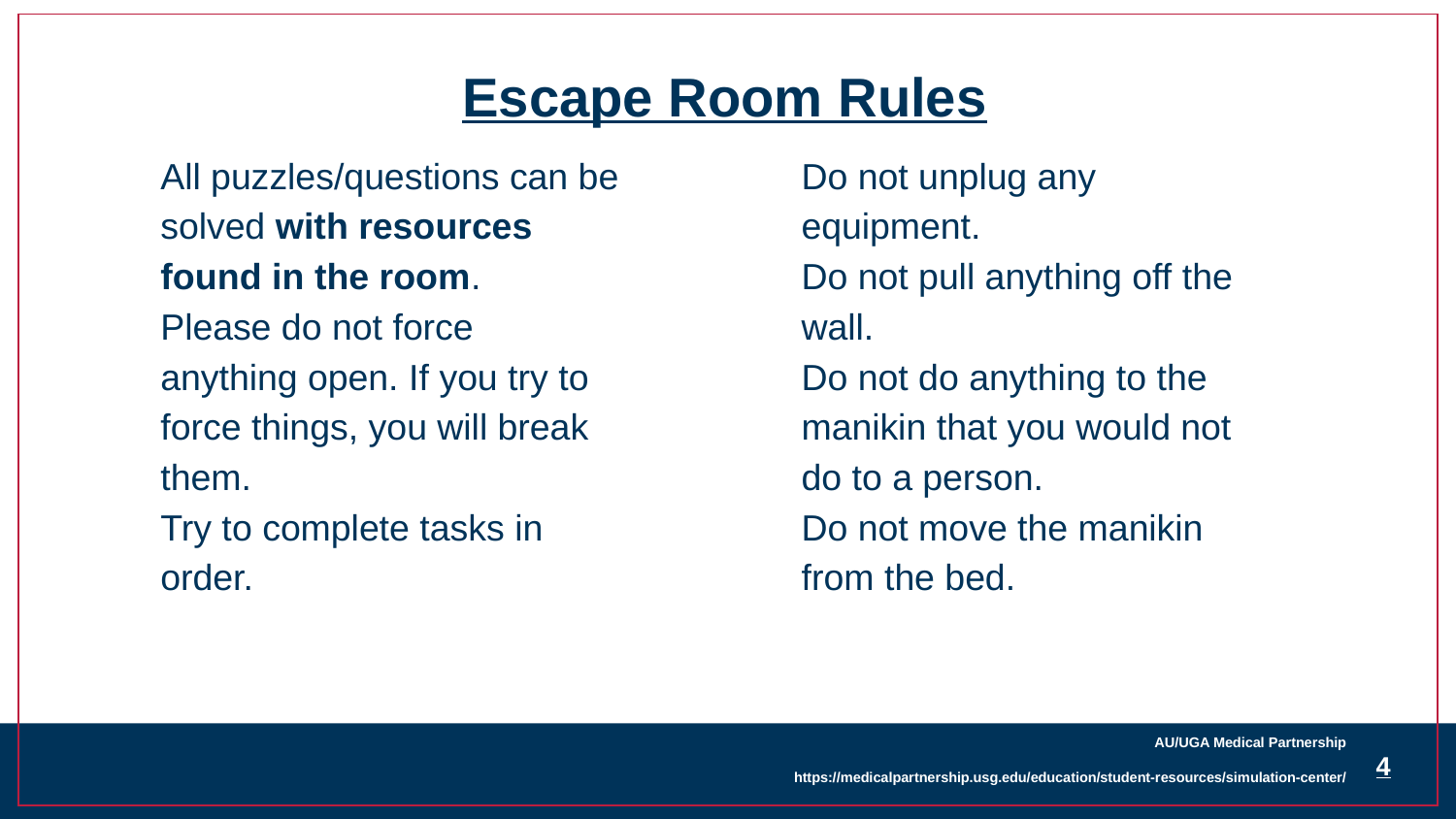

Escape Room Rules
All puzzles/questions can be solved with resources found in the room.
Please do not force anything open. If you try to force things, you will break them.
Try to complete tasks in order.
Do not unplug any equipment.
Do not pull anything off the wall.
Do not do anything to the manikin that you would not do to a person.
Do not move the manikin from the bed.
AU/UGA Medical Partnership
https://medicalpartnership.usg.edu/education/student-resources/simulation-center/

## Slide 5
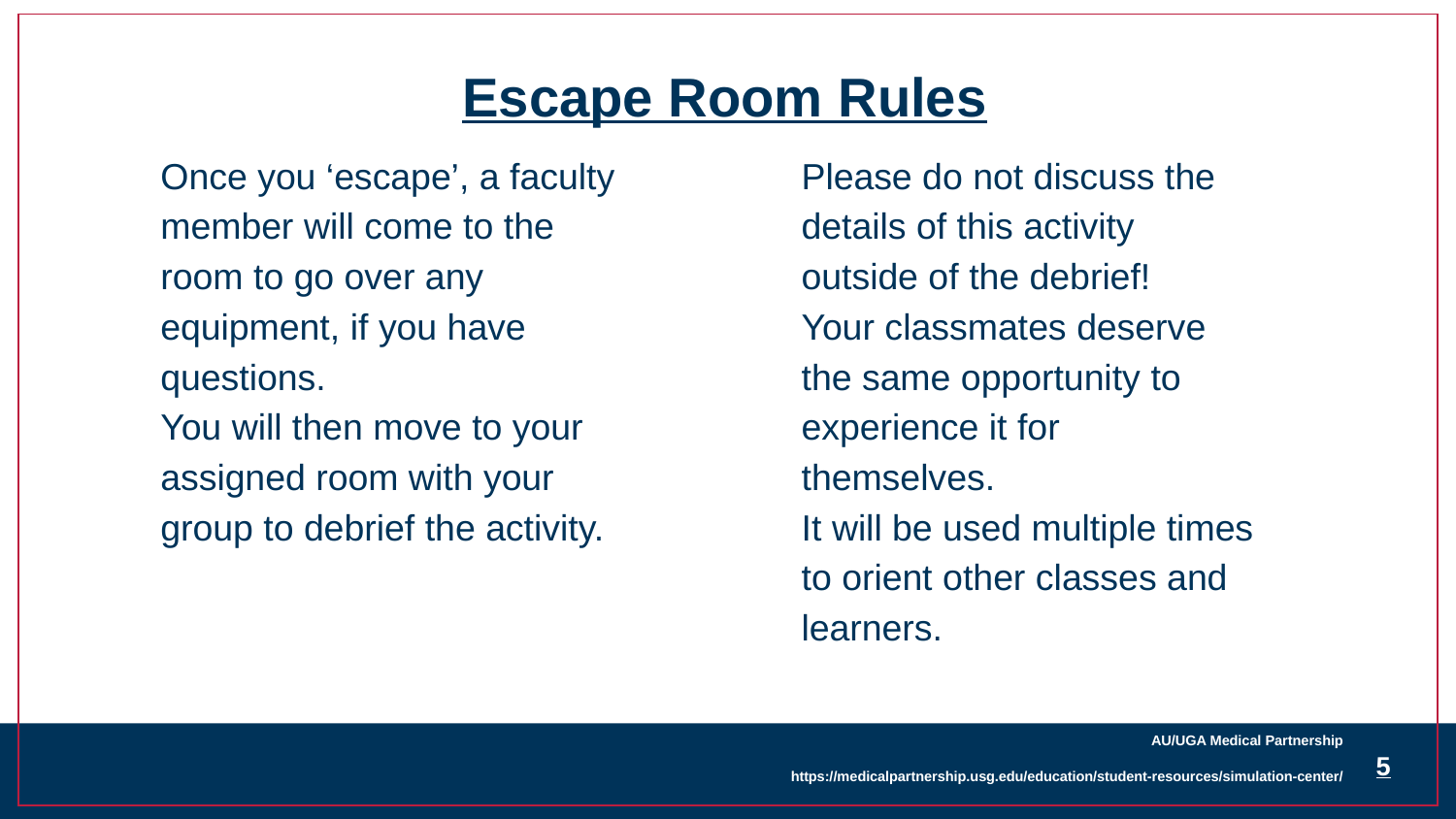

Escape Room Rules
Please do not discuss the details of this activity outside of the debrief!
Your classmates deserve the same opportunity to experience it for themselves.
It will be used multiple times to orient other classes and learners.
Once you ‘escape’, a faculty member will come to the room to go over any equipment, if you have questions.
You will then move to your assigned room with your group to debrief the activity.
AU/UGA Medical Partnership
https://medicalpartnership.usg.edu/education/student-resources/simulation-center/

## Slide 6
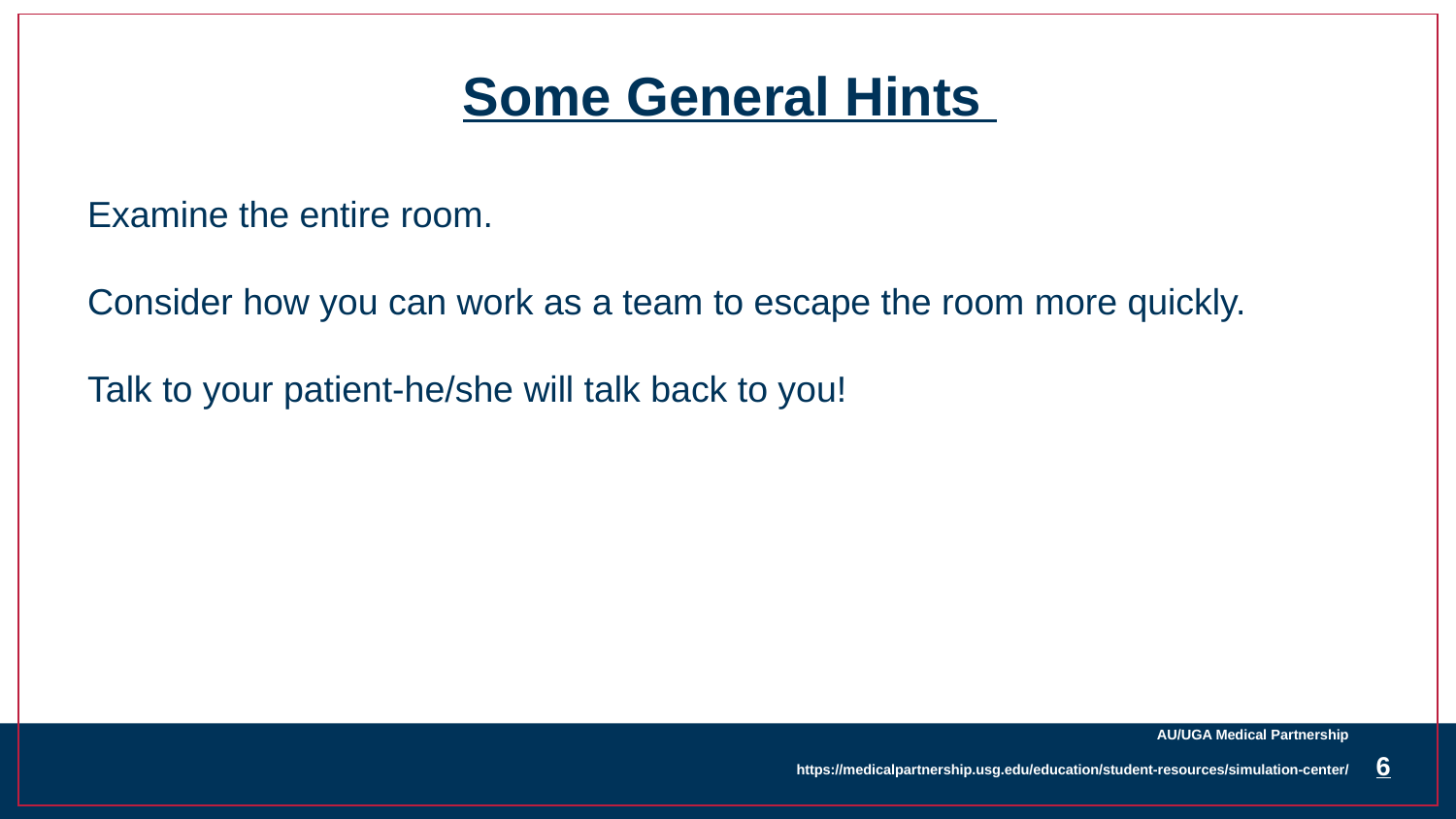

Some General Hints
Examine the entire room.
Consider how you can work as a team to escape the room more quickly.
Talk to your patient-he/she will talk back to you!
AU/UGA Medical Partnership
https://medicalpartnership.usg.edu/education/student-resources/simulation-center/

## Slide 7
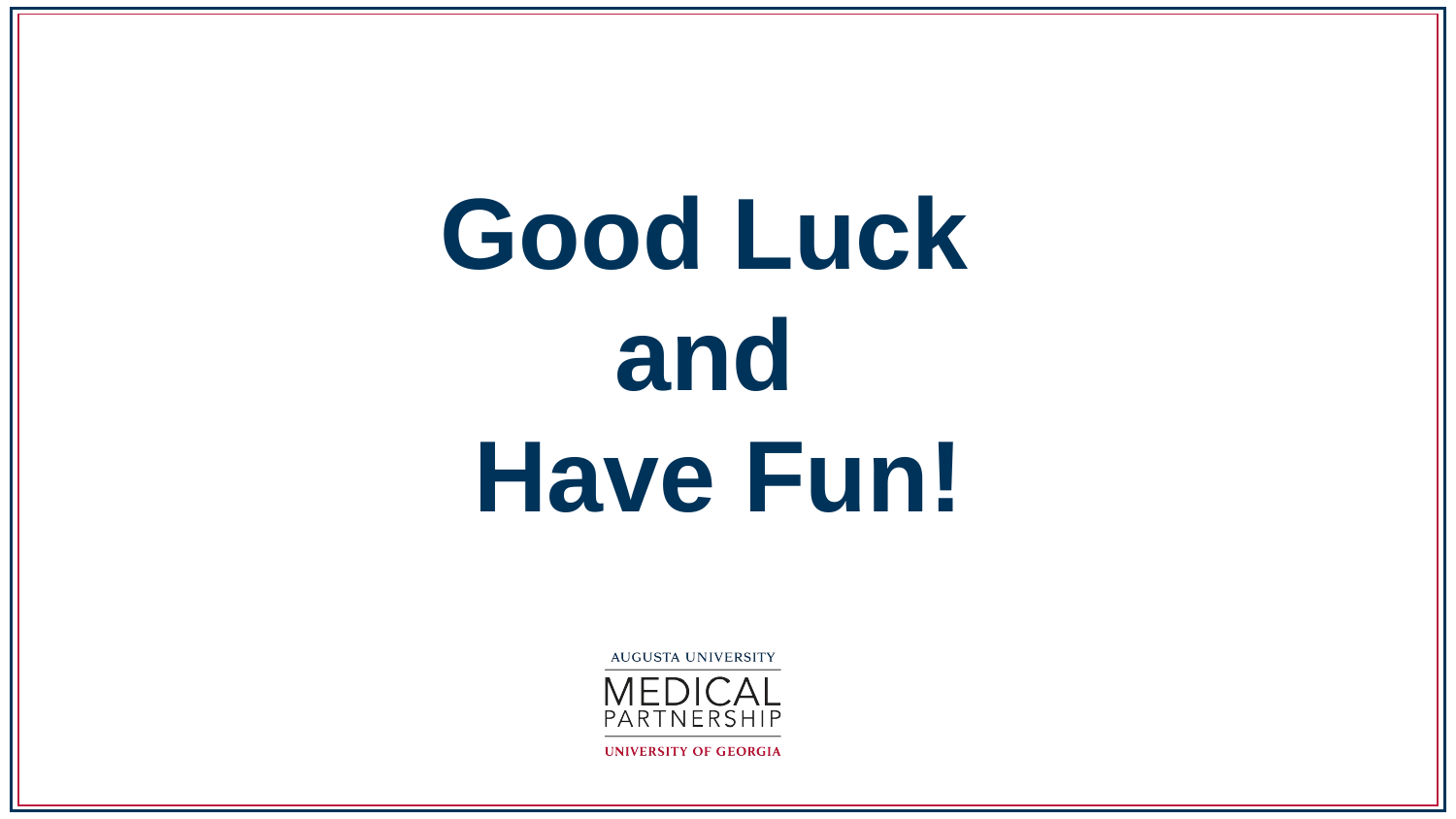

Good Luck
and
Have Fun!
