# Supplementary material for: An Escape Room to Orient Preclinical Medical Students to the Simulated Medical Environment
Source: MedEdPORTAL. 2022 Mar 25;18:11229. doi: 10.15766/mep_2374-8265.11229 (PMC8948100; doi:10.15766/mep_2374-8265.11229)
Supplement: Supplementary file 1 — Escape Room Simulation Guide.docxRoom Layout.pdfPatient Chart and Puzzle Template.pdfClue and Exam Findings Cards.pdfAdditional Room Resources.docxParticipant Prebriefing.pptxEscape Room Flow Chart and Codes.pdfExit Questionnaire.docxFaculty Instructions and Debriefing Guidelines.pdfCritical Actions Checklist.docxParticipant Evaluation.docxFollow-up Survey.docx [file mep_2374-8265.11229-s001.zip › Educational_Summary_Report_11229.pdf]

MedEdPORTAL® The AAMC Journal of Teaching and Learning Resources

Original Publication Open Access

An Escape Room to Orient Preclinical Medical Students to the Simulated Medical Environment

Aimee Martin, MD*, Sarah Gibbs, MS

*Corresponding Author: aimeem@uga.edu

Abstract

Introduction: Simulation is increasingly being used in the preclinical years of US medical school curricula to provide experiential learning opportunities for students. However, preclinical medical students may not be able to access the full benefits of immersive simulation scenarios without an in-depth introduction to the simulation environment and manikin. An escape room may be an effective way to orient students in an interactive manner to overcome this barrier. **Methods:** We designed and implemented a 90-minute escape room orientation activity to address student discomfort in the simulation environment by providing a team-based, hands-on exploration of identified critical features of the room and manikin in the guise of a routine clinic visit for a patient. We surveyed learners on their confidence immediately following the escape room and on their perceptions of the session effectiveness following their first simulation. **Results:** A total of 148 preclinical medical students participated in the escape room activity in 30 groups of four to five persons. Of those students, 130 participated in a simulated patient case within 1 month of the escape room activity, and 89 filled out a follow-up survey. Of responding students, 80% reported that the escape room activity was *highly effective* or *very effective* in preparing them for participation in a simulated patient case. **Discussion:** Implementing an escape room orientation activity for preclinical medical students was effective in preparing students to participate in their first immersive simulation scenario.

Keywords
Escape Room, Communication Skills, Educational Technology, Games, Simulation

Educational Objectives

By the end of this activity, learners will be able to:

1. Recall the layout of the simulation patient bay.
2. Demonstrate the use of basic equipment in the simulation bay.
3. Identify basic features of the simulation manikin.

Introduction

Simulation is increasingly being used in the preclinical years of US medical school curricula[1] to provide experiential learning opportunities for students. The use of simulation-based education in health care is recognized as an effective teaching methodology that improves student outcomes.[2] Simulation best practices describe prebriefing prior to a simulation activity as a key component of preparing a student for a successful learning experience. Prebriefing should include an orientation to the simulation environment and equipment that is student centered and appropriate to the level of learning and competency.[3,4] A thorough orientation to simulation prior to participating in simulated cases sets up learners for successful navigation of the environment, the technology, and the processes, making them more likely to engage in the activity.[5] Currently, the extent and nature of the orientation to the simulation environment are not prescribed, but the level of the student should be taken into consideration. For example, preclinical students require a more in-depth experience to ensure a safe and effective learning environment for simulations, in contrast to postgraduate learners, who are already familiar with clinical environments and comfortable with patient care.[6,7]

Citation:
Martin A, Gibbs S. An escape room to orient preclinical medical students to the simulated medical environment. *MedEdPORTAL*. 2022;18:11229. https://doi.org/10.15766/mep_2374-8265.11229

Preclinical medical students in the first and second years of their education learn the basic components of a physical exam but have limited opportunities to hone those skills and become comfortable with the components of a typical exam on a person. Thus, students may have difficulty both performing an exam on a manikin and interpreting findings as typical or atypical. They are still learning the art of obtaining a history and creating

Copyright © 2022 Martin and Gibbs This is an open-access publication distributed under the terms of the Creative Commons Attribution license.

rapport with patients and can find the simulated patient manikins especially intimidating, as manikins do not display the expected facial expressions or physical mannerisms of a person. Students also have limited exposure to patient rooms, patient monitors, and other equipment in clinical care settings. When preclinical simulation activities place students in the simulated patient environment without a thorough orientation, there can be confusion, uncertainty, and a sense of not belonging in the space, which can prevent students from fully investing in the learning experience.[8]

Feedback from students participating in our institution's simulation curriculum indicated a desire for a more detailed orientation to the simulation environment and manikin, with more attention to the physiologic features of the manikin and the location and use of equipment in the room. The former orientation consisted of an interactive online module. We hypothesized that an in-person orientation would be beneficial to students prior to participating in the simulation curriculum, and we felt that a self-directed, gamified experience would provide the most effective method of instruction.

Escape rooms are becoming popular in simulation-based education, and they are similarly grounded in accepted educational theories for adult students. Escape rooms provide concrete experiences that allow for reflective observation and active experimentation, as detailed in Kolb's experiential learning theory.[9] They also appeal to adult students' focus on problem-solving and desire for interactive learning.[10-13] Health care simulation escape rooms engage students in an interactive activity that incorporates clues and puzzle solving with health care-related themes.[10,14-17] Escape rooms such as Diemer and colleagues' patient safety escape room,[18] published in *MedEdPORTAL,* provide an opportunity to deliver both formative and summative training in technical and nontechnical skills. They have also become more commonly used in medical education over the past 3 years.[14-28] Other escape rooms that have been described in the literature are of varied design, ranging from immersive[20,21] to tabletop[22] to virtual.[23]

We created an interactive escape room game to allow students to explore the simulated patient room and the simulated patient manikin. Our escape room is unique from other escape rooms described in the literature in that it is used as an interactive orientation to the simulation environment rather than primarily as a treatment-focused patient case. During development of this case, we were unable to find a comparable simulation orientation activity designed as an escape room for medical students with detailed setup and operation instructions. However, while our resource was under review, an escape room used as an orientation for nursing students was published.[28] Our activity can be used as a part of prebriefing exercises to allow preclinical students to become oriented to the simulation space and to the logistics of a simulation in an enjoyable and interactive fashion, and it can provide a solid foundation from which to begin participation in patient care scenarios.

Methods

Development

The escape room was designed for preclinical medical students with the expectation that they would have learned the skills of basic vital sign measurement and limited physical exam maneuvers including auscultation of the heart and lungs; examination of the abdomen; abbreviated head, eyes, ears, nose, and throat examination; and neurological examination. We first identified key tasks that students would have to perform when participating in patient simulation scenarios. We then created a simple backstory, a patient presenting for a routine primary care clinic visit, that required students to perform the identified tasks sequentially to complete the patient visit and escape the clinic room. The tasks included performing hand hygiene, obtaining vital signs, and performing a brief physical exam on the patient using basic equipment in the room. These tasks provided the context to introduce the equipment and the physiologic functions of the patient manikin in a hands-on, interactive manner.

We piloted the case on three separate occasions with groups of two or three faculty members or medical students in their clinical years of training. We incorporated feedback from these groups into the development of the activity. We also incorporated feedback from faculty after each group of students completed the activity, resulting in slight modifications.

Equipment/Environment

The escape room activity took place in a simulation suite with simulated patient bays outfitted identically with a patient monitor, bedside table, rolling stool, table, and crash cart (see Appendix A for the simulation guide). We used five patient rooms, which allowed five groups to participate in the activity simultaneously. The rooms were reset after each session. The list and location of items needed for room setup could be viewed in the room layout document (Appendix B). Appendices C, D, and E provided printable documents and cards needed for room setup. The Basic Life Support algorithm[29] and the Blood Pressure Categories chart[30] used as additional resources needed to be downloaded from the American Heart Association and placed in the room as indicated in Appendix B.

Copyright © 2022 Martin and Gibbs This is an open-access publication distributed under the terms of the Creative Commons Attribution license.

Personnel
Facilitators included faculty clinician and basic science educators who taught in the first 2 years of the medical school curriculum and who had also taught in the simulation curriculum the prior year. They had been previously oriented to the simulation bay and manikins. For this activity, facilitators were required to attend either a 1-hour virtual guided tour of the escape room via a videoconferencing application that allowed real-time viewing of the tour or an in-person guided tour. They were also provided with a copy of the escape room appendices, which were reviewed with them in detail. Faculty were given the opportunity to ask questions and receive clarification during both tour options. No previous experience with an escape room was required.

One facilitator provided a large-group prebriefing immediately prior to the start of the escape room activity. Then, each escape room required one facilitator per student group to act as the remote manikin operator, observe the activity, record the group completion time, record the performance of critical actions, and facilitate the small-group debriefing. The simulation coordinator and an assistant were present to help with setup, resetting rooms, equipment troubleshooting, and other logistical details.

Implementation
Students were provided with a link to an online introductory module that had previously been used for orientation and were advised that completion of the module was required prior to participating in the escape room, though completion of the module was not verified. We divided 148 students into 30 groups, composed of four to five students per group. We used five patient rooms, which allowed five groups to take part in the activity simultaneously. Students participated in the escape room a few weeks prior to their first simulated patient case scenario.

Immediately prior to starting the escape room, all students attended an in-person, 10-minute prebriefing (Appendix F) that provided the session logistics, objectives, backstory, and rules. Students were not permitted to bring any personal electronic devices, such as tablets or cell phones, with them to the activity (a rule stated in the prebriefing). Learner groups then proceeded to the simulated patient bay, and once they were in the room, the clock was started. Faculty used Appendix A, containing a detailed description of the ideal progression of students through the escape room, and Appendix G, featuring a step-by-step sequence of the game with puzzle solutions, scripted hints, and responses.

The final step of the escape room activity was the completion of an exit questionnaire (Appendix H) that was accessed with the tablet camera via a QR code on the patient's abdomen. Once completed, the message "Congratulations! You have completed your patient's clinic visit in time and have escaped the room!" was displayed, and the activity ended. Groups had 40 minutes to escape the room; then, students attended a small-group debriefing session with the facilitator.

Debriefing
Immediately following completion of the activity, each group participated in a 40-minute debriefing, facilitated by the observing faculty member. Faculty used the PEARLS debriefing tool,[31,32] the faculty instructions and debriefing guidelines (Appendix I), and the completed critical actions checklist (Appendix J) to help initiate discussion. Debriefing focused on reviewing the layout of the simulation patient bay, use of the equipment, and features of the manikin. We provided debriefing prompts to encourage discussion of the available resources in the room and the experience of interacting with the patient manikin, as well as to reinforce the physiologic functions of the manikin. Appendix I included key debrief points and information to assist in answering questions that might arise regarding capabilities of the manikin, room equipment, and patient monitor.

We allowed faculty to spend 10 minutes in the room with students, if needed, to go over any equipment or manikin functions that remained unclear to the learners prior to moving to a separate debriefing space. While groups finished debriefing, Simulation Center staff members reset the rooms, placing all pieces back in their designated locations according to the room layout document. Room reset took 10 minutes. All clue and exam findings cards (Appendices C-E) were laminated so they could be written on with dry-erase markers and easily erased.

Assessment
Twelve tasks were built into the escape room sequence and detailed in a critical actions checklist (Appendix J). As they observed the students complete the tasks, faculty filled out the checklist, which served as the assessment of Educational Objectives 1 and 3. These tasks included using equipment in the patient rooms that the students would frequently interact with, or typically have difficulty with, as well as selected patient manikin features used routinely in simulations. Learners had 40 minutes to complete all of the tasks and escape the room. The time to escape the room was the base score. One minute was added to their time for each hint they received (whether solicited or not). One minute was added for each critical action item they did not complete. The lowest score indicated the fastest group.

Copyright © 2022 Martin and Gibbs This is an open-access publication distributed under the terms of the Creative Commons Attribution license.

Achievement of Educational Objective 2 was assessed during the debriefing.

Two surveys (Appendices K and L), developed using the DASH[33] and SET-M[34] as a framework, were given to students to evaluate the effectiveness of the escape room activity in meeting curricular needs. The first survey was administered immediately after the debriefing of the escape room activity. The survey asked students to rate their comfort level in performing the 12 tasks on the critical actions checklist and to provide feedback on the activity, including the prebriefing and the facilitated debriefing, as well as to suggest improvements.

The second survey, administered immediately after students had completed their first simulated patient case 3 weeks later, queried them on whether the escape room activity had been effective in preparing them for participating in a simulated patient case in the Simulation Center.

Results

A total of 148 preclinical medical students participated in our escape room orientation. Of the 30 groups, 29 were able to complete all 12 critical actions and escape the room. The fastest escape time was 26 minutes and 49 seconds, with an average time of 37 minutes and 44 seconds, including time penalties. One group, due to a misunderstanding of a faculty-provided hint, abandoned solving the puzzles in favor of just completing a physical exam of the manikin, thereby never advancing to interacting with the patient monitor or discovering the locked stethoscope necessary for obtaining a manual blood pressure and for auscultation.

Immediately after completion of the escape room activity, 107 participants completed a voluntary, anonymous evaluation rating their confidence in their abilities to interact with the equipment in the room (Table 1). The median response for all critical actions was 4 (out of 5), except for "perform hand hygiene in the simulated patient room" (*Mdn* = 5) and "obtain a manual blood pressure on the patient simulator" (*Mdn* = 3).

Learner feedback was overwhelmingly positive, with comments including the following:

- "I like that we worked together and got oriented to the room in a very engaging way."
- "I most enjoyed the opportunity to become oriented with the simulation room by an interactive means, in the form of a puzzle."
- "It was a great situation to think on your feet and work with teammates to find solutions."

Table 1. Learner Confidence in Critical Actions (*N* = 107)

Critical Action[a]	*Mdn*
Perform hand hygiene in the simulated patient room.	5
Adjust the patient bed.	4
Communicate with the patient simulator.	4
Use the in-room phone.	4
Locate the Basic Life Support algorithm in the room.	4
Read the patient vital signs on the monitor.	4
Obtain a manual pulse on the patient simulator.	4
Obtain a manual blood pressure on the patient simulator.	3
Obtain an automated blood pressure on the patient monitor.	4
Examine the patient simulator's pupils.	4
Examine the patient simulator's heart and lungs.	4
Examine the patient simulator's abdomen.	4

[a]Rated on a 5-point Likert scale (1 = *not confident,* 2 = *slightly confident,* 3 = *moderately confident,* 4 = *very confident,* 5 = *extremely confident*).

- "A fun introduction! I feel confident about going into a sim room now."

A total of 130 preclinical medical students participated in a simulated patient case approximately 3 weeks after completing the escape room activity. Immediately after the patient case, 89 participants completed a voluntary, anonymous, follow-up evaluation rating the effectiveness of the escape room in acclimating them to the simulated environment (Table 2). Eighty-two percent of respondents rated the escape room as *highly effective* or *very effective* in preparing them for future simulated patient scenarios, and 80% felt it was *highly effective* or *very effective* in acclimating them to the simulated patient manikin.

Discussion

The escape room activity successfully met our goal of introducing our preclinical medical students to the simulation manikin and environment in a fun, interactive, and low-stakes manner. The activity provided a foundation for students to work from during future simulated patient cases. Our resource is unique in its use of an escape room to provide a hands-on and experiential orientation to simulation for learners. This activity could prove to be eminently useful in many health care professions training settings as simulation gains popularity in health care education. Creating an escape room activity was time consuming, and

Table 2. Escape Room Effectiveness (*N* = 89)

Query[a]	*Mdn*
Did you find the escape room to be effective in preparing you for participating in a simulated patient scenario in the Simulation Center?	4
Did you find the escape room to be effective for acclimating you to the simulated patient room?	4
Did you find the escape room to be effective for acclimating you to the simulated patient manikin?	5

[a]Rated on a 5-point Likert scale (1 = *not effective,* 2 = *somewhat effective,* 3 = *moderately effective,* 4 = *very effective,* 5 = *highly effective*).

Copyright © 2022 Martin and Gibbs This is an open-access publication distributed under the terms of the Creative Commons Attribution license.

finding detailed instructions on the design, setup, and operation of escape room activities targeting medical student learning was challenging. We hope that providing this detailed resource will fill a gap and benefit programs using simulation in the preclinical years of medical school.

Lessons Learned

We followed the International Nursing Association of Clinical and Simulation Learning's Standards of Best Practice: Simulation Design[4] when developing this activity by providing a participant prebriefing and following the activity with a debriefing. While the escape room activity is essentially part of a larger prebriefing to simulation, we would like to emphasize the need for a prebriefing for the escape room itself in order to establish the learning objectives, logistics, and ground rules. These elements provide students with the added benefit of an introduction to the logistics and flow of future simulations. Most of our students indicated in an informal poll that they had never participated in a health care simulation exercise or in an escape room. We did not find that a lack of experience with escape rooms was a detractor to this experience, as the clues that we provided satisfactorily guided students through the exercise and a method for faculty to provide hints was available.

Our students did experience some discomfort upon having the manikin speak to them, finding it initially confusing to be both in a game and attending to a patient. Students requested that they be informed that the manikin would speak to them and should be addressed as a patient whom they were helping through a clinic visit, which was amended in the prebriefing presentation. We also revised the wording of the hints so as to allow faculty to maintain their role as the patient while providing the hints.

During piloting, we found that several clues were able to be skipped or accessed out of sequence, which allowed students to advance while missing some critical actions. This underlines the importance of testing linking clues and preventing early reveals of clues.[35,36] Additional lockboxes with associated clues and puzzles resolved these issues.

We noted that many groups had difficulty in measuring a manual blood pressure, as indicated by the lower confidence score for this task. We suspect this was due to several reasons, including that the task could only be addressed by a single student at a time, preventing others from having the opportunity to attempt it. Learner inexperience with blood pressure measurement, as well as inherent limitations of the manikin (e.g., blood pressure could only be taken from the left arm), could have also contributed to the lower confidence scores. To address this, we added a hint allowing operators to provide the blood pressure if needed. Students also struggled with answering a question regarding the lowest normal systolic blood pressure despite having a blood pressure chart. We opted to retain the original wording of the question despite the difficulty it caused, as it required learners to pay attention to detail, a valuable skill in clinical practice.

We found that due to the time incentive and the team-based nature of the activity, not every participant was involved in solving every clue or performing every critical action, resulting in some participants not gaining satisfactory comfort levels with some of the tasks. We addressed this with our later groups by instructing faculty to provide a short, in-room debrief prior to the more formal small-group debriefing session so that students would have the opportunity to ask for clarification or demonstration of the equipment. Finally, one group failed to complete all of the critical actions and did not escape the room. This limited the usefulness of the activity, as the goal was to have all students complete the room in a particular order to become familiar with the items in the room. We provided more suggested hints and prompts for faculty to use to keep groups on track to completing the activity.

Limitations

Our results were limited by a low response rate to the second questionnaire, likely because it was voluntary. There are several barriers such as class size, facilities, and time that other institutions might face when implementing this case. We have a small class size and a large number of faculty with time to help with simulation activities. We also have enough manikins and simulation stations to facilitate a small student-to-manikin ratio. Additionally, our curricular leadership allotted curricular time for this activity.

Conclusions

We successfully used an escape room format to orient preclinical medical students to the manikin and simulation environment. Students indicated confidence in using basic equipment in the room and assessing manikin vital signs after completion of the escape room, and they found the activity very effective in preparing them to participate in simulated patient encounters. While not a primary objective, we also found the activity useful in initiating discussion about teamwork in the debriefing session. A group that works together as a well-functioning team can escape the room in less time than a group with poor organization and a lack of teamwork skills. Students commented on how their communication and situational awareness affected their performance as a group. This can provide a lead-in for future training in teamwork through health care simulation exercises.

Copyright © 2022 Martin and Gibbs This is an open-access publication distributed under the terms of the Creative Commons Attribution license.

This activity could be adapted to orient students in other health care professions by altering the critical actions. For more advanced students, substitution of clues requiring more medical knowledge or changing the background scenario to an emergent case would suffice. For programs that do not need this type of orientation, the learning objectives could be modified to focus the activity on developing teamwork.

Appendices

A. Escape Room Simulation Guide.docx
B. Room Layout.pdf
C. Patient Chart and Puzzle Template.pdf
D. Clue and Exam Findings Cards.pdf
E. Additional Room Resources.docx
F. Participant Prebriefing.pptx
G. Escape Room Flow Chart and Codes.pdf
H. Exit Questionnaire.docx
I. Faculty Instructions and Debriefing Guidelines.pdf
J. Critical Actions Checklist.docx
K. Participant Evaluation.docx
L. Follow-up Survey.docx

All appendices are peer reviewed as integral parts of the Original Publication.

Aimee Martin, MD: Clinical Assistant Professor and Campus Director of Simulation and Ultrasound, Augusta University and University of Georgia Medical Partnership; ORCID: https://orcid.org/0000-0001-5384-6294

Sarah Gibbs, MS: Coordinator, Simulation Center, Augusta University and University of Georgia Medical Partnership

Acknowledgments
Special thank you to Jennifer Stowe, Augusta University and University of Georgia (AU/UGA) Medical Partnership Medical Illustrator and Graphic Designer, for her invaluable assistance in designing and formatting the appendices documents; to Julie Gaines, MLIS, AU/UGA Medical Partnership Library Director, for her expert assistance with editing the manuscript and reviewing references; and to Dr. Gerald Crites for his comments on the manuscript.

Disclosures
None to report.

Funding/Support
None to report.

Prior Presentations
Martin A, Gibbs S, Bassett C, Gaines J, Boegehold M. Using an escape room activity to orient learners to the simulated medical environment. Presented virtually at: International Association of Medical Science Educators Conference; June 12-15, 2021.

Ethical Approval
Reported as not applicable.

References

1. Passiment M, Sacks H, Huang G. *Medical Simulation in Medical Education: Results of an AAMC Survey*. Association of American Medical Colleges; 2011. https://store.aamc.org/medical-simulation-in-medical-education-results-of-an-aamc-survey-pdf.html
2. McGaghie WC, Issenberg SB, Petrusa ER, Scalese RJ. Effect of practice on standardised learning outcomes in simulation-based medical education. *Med Educ.* 2006;40(8):792-797. https://doi.org/10.1111/j.1365-2929.2006.02528.x
3. INACSL Standards Committee. INACSL Standards of Best Practice: Simulation facilitation. *Clin Simul Nurs*. 2016;12(S):S16-S20. https://doi.org/10.1016/j.ecns.2016.09.007
4. INACSL Standards Committee. INACSL Standards of Best Practice: Simulation simulation design. *Clin Simul Nurs*. 2016;12(S):S5-S12. https://doi.org/10.1016/j.ecns.2016.09.005
5. Rudolph JW, Raemer DB, Simon R. Establishing a safe container for learning in simulation: the role of the presimulation briefing. *Simul Healthc*. 2014;9(6):339-349. https://doi.org/10.1097/SIH.0000000000000047
6. Wang E, Kharasch M, Aitchison P, et al. Simulation in health care, part I: the future of medical education and training. *Dis Mon*. 2011;57(11):657-756.
7. Rutherford-Hemming T, Lioce L, Breymier T. Guidelines and essential elements for prebriefing. *Simul Healthc*. 2019;14(6): 409-414. https://doi.org/10.1097/SIH.0000000000000403
8. Fraser KL, Ayres P, Sweller J. Cognitive load theory for the design of medical simulations. *Simul Healthc*. 2015;10(5): 295-307. https://doi.org/10.1097/SIH.0000000000000097
9. Kolb DA. *Experiential Learning: Experience as the Source of Learning and Development*. 2nd ed. Pearson Education; 2014.
10. Backhouse A, Malik M. Escape into patient safety: bringing human factors to life for medical students. *BMJ Open Qual*. 2019;8(1):e000548. https://doi.org/10.1136/bmjoq-2018-000548
11. Hamari J, Shernoff DJ, Rowe E, Coller B, Asbell-Clarke J, Edwards T. Challenging games help students learn: an empirical study on engagement, flow and immersion in game-based learning. *Comput Human Behav*. 2016;54:170-179. https://doi.org/10.1016/j.chb.2015.07.045

Copyright © 2022 Martin and Gibbs This is an open-access publication distributed under the terms of the Creative Commons Attribution license.

12. Zigmont JJ, Kappus LJ, Sudikoff SN. Theoretical foundations of learning through simulation. *Semin Perinatol*. 2011;35(2):47-51. https://doi.org/10.1053/j.semperi.2011.01.002
13. Brannan JD, White A, Bezanson JL. Simulator effects on cognitive skills and confidence levels. *J Nurs Educ*. 2008;47(11):495-500. https://doi.org/10.3928/01484834-20081101-01
14. Brown N, Darby W, Coronel H. An escape room as a simulation teaching strategy. *Clin Simul Nurs.* 2019;30:1-6. https://doi.org/10.1016/j.ecns.2019.02.002
15. Adams V, Burger S, Crawford K, Setter R. Can you escape? Creating an escape room to facilitate active learning. *J Nurses Prof Dev.* 2018;34(2):E1-E5. https://doi.org/10.1097/NND.0000000000000433
16. *Escape Room Guide.* University of Kansas Health System: date unknown. https://www.kansashealthsystem.com/-/media/Files/PDF/Nursing-Escape-Room-guide-update-_final.pdf
17. Anderson M, Lioce L, Robertson JM, Lopreiato JO, Díaz DA. Toward defining health care simulation escape rooms. *Simul Gaming*. 2021;52(1):7-17. https://doi.org/10.1177/1046878120958745
18. Diemer G, Jaffe R, Papanagnou D, Zhang XC, Zavodnick J. Patient safety escape room: a graduate medical education simulation for event reporting. *MedEdPORTAL*. 2019;15:10868. https://doi.org/10.15766/mep_2374-8265.10868
19. Valdes B, Mckay M, Sanko JS. The impact of an escape room simulation to improve nursing teamwork, leadership and communication skills: a pilot project. *Simul Gaming*. 2021;52(1):54-61. https://doi.org/10.1177/1046878120972738
20. Rosenkrantz O, Jensen TW, Sarmasoglu S, et al. Priming healthcare students on the importance of nontechnical skills in healthcare: how to set up a medical escape room game experience. *Med Teach*. 2019;41(11):1285-1292. https://doi.org/10.1080/0142159X.2019.1636953
21. Morrell B, Eukel HN. Shocking escape: a cardiac escape room for undergraduate nursing students. *Simul Gaming*. 2021;52(1):72-78. https://doi.org/10.1177/1046878120958734
22. Guckian J, Eveson L, May H. The great escape? The rise of the escape room in medical education. *Future Healthc J*. 2020;7(2):112-115. https://doi.org/10.7861/fhj.2020-0032
23. Dacanay AP, Sibrian J, Wyllie C, Sorrentino E, Dunbar G. Can you escape sepsis? Using a healthcare escape room as an innovative approach to nursing education. *Clin Nurse Spec*. 2021;35(2):65-72. https://doi.org/10.1097/NUR.0000000000000578
24. Smith MM, Davis RG. Can you escape? The pharmacology review virtual escape room. *Simul Gaming*. 2021;52(1):79-87. https://doi.org/10.1177/1046878120966363
25. Sanders JE, Kutzin J, Strother CG. Escape the simulation room. *Simul Gaming*. 2021;52(1):62-71. https://doi.org/10.1177/1046878120963591
26. Roman P, Rodriguez-Arrastia M, Molina-Torres G, Márquez-Hernández VV, Gutiérrez-Puertas L, Ropero-Padilla C. The escape room as evaluation method: a qualitative study of nursing students' experiences. *Med Teach*. 2020;42(4):403-410. https://doi.org/10.1080/0142159X.2019.1687865
27. Kutzin JM. Escape the room: innovative approaches to interprofessional education. *J Nurs Educ*. 2019;58(8):474-480. https://doi.org/10.3928/01484834-20190719-07
28. Robertiello G, Genee J, Marrera A. Escape the sim! An escape room innovation to orient learners to the simulation environment. *Nurs Educ Perspect*. 2021;42(3):195-196. https://doi.org/10.1097/01.NEP.0000000000000585
29. Olasveengen TM, Mancini ME, Perkins GD, et al; Adult Basic Life Support Collaborators. Adult basic life support: international consensus on cardiopulmonary resuscitation and emergency cardiovascular care science with treatment recommendations. *Resuscitation*. 2020;156:A35-A79. https://doi.org/10.1016/j.resuscitation.2020.09.010
30. Whelton PK, Carey RM, Aronow WS, et al. 2017 ACC/AHA/AAPA/ABC/ACPM/AGS/APhA/ASH/ASPC/NMA/PCNA guideline for the prevention, detection, evaluation, and management of high blood pressure in adults: a report of the American College of Cardiology/American Heart Association Task Force on Clinical Practice Guidelines. *J Am Coll Cariol*. 2018; 71(19):e127-e248. https://doi.org/10.1016/j.jacc.2017.11.006
31. Eppich W, Cheng A. Promoting Excellence And Reflective Learning in Simulation (PEARLS): development and rationale for a blended approach to health care simulation debriefing. *Simul Healthc*. 2015;10(2):106-115. https://doi.org/10.1097/SIH.0000000000000072
32. Bajaj K, Meguerdichian M, Thoma B, Huang S, Eppich W, Cheng A. The PEARLS health care debriefing tool. *Acad Med*. 2018; 93(2):336. https://doi.org/10.1097/ACM.0000000000002035
33. *Debriefing Assessment for Simulation in Healthcare (DASH) Student Version*. Center for Medical Simulation; 2010. https://harvardmedsim.org/wp-content/uploads/2017/01/DASH.SV.Long.2010.Final.pdf
34. Leighton K, Ravert P, Mudra V, Macintosh C. Updating the Simulation Effectiveness Tool: item modifications and reevaluation of psychometric properties. *Nurs Educ Perspect*. 2015;36(5):317-323. https://doi.org/10.5480/15-1671
35. Eukel H, Morrell B. Ensuring educational escape-room success: the process of designing, piloting, evaluating, redesigning, and re-evaluating educational escape rooms. *Simul Gaming*. 2021;52(1):18-23. https://doi.org/10.1177/1046878120953453
36. Davis K, Lo H, Lichliter R, et al. Twelve tips for creating an escape room activity for medical education. *Med Teach*. Published online April 19, 2021. https://doi.org/10.1080/0142159X.2021.1909715

Received: April 7, 2021
Accepted: December 29, 2021
Published: March 25, 2022

Copyright © 2022 Martin and Gibbs This is an open-access publication distributed under the terms of the Creative Commons Attribution license.
